# Capacity for upregulation of emotional processing in psychopathy: all you have to do is ask

**DOI:** 10.1093/scan/nsy088

**Published:** 2018-09-25

**Authors:** Matthew S Shane, Lindsay L Groat

**Affiliations:** 1 University of Ontario Institute of Technology, Forensic Psychology Program, 2000 Simcoe St. N, Oshawa, ON, Canada; 2 The Mind Research Network, Albuquerque, NM, USA

**Keywords:** psychopathy, emotion, emotion regulation, upregulation, downregulation, fMRI

## Abstract

Historically, psychopathic individuals have been described as suffering a chronic hyporesponsivity to negatively valent stimuli. However, while a wide body of empirical work indicates that the psychopath does not manifest normal reactivity to emotional stimuli, it does not similarly indicate that they cannot do so. To attempt to differentiate these alternatives, the current functional magnetic resonance imaging (fMRI) study evaluated the extent to which offenders with varying PCL-R scores could up- (or down-) regulate their neural response to negatively valent stimuli. Participants were asked to either watch negatively- and neutrally-valent images naturally (passive-processing), or to try to increase or decrease their emotional response to the images (instructed-processing). During passive processing, high-psychopathy offenders showed reduced activity compared to both low- and mid-psychopathic offenders through a majority of emotion-relevant regions. However, when participants were instructed to try to increase their emotional response all groups showing increased activity throughout relevant regions, including left insula, orbitofrontal cortex and anterior cingulate/medial frontal cortex (ACC/mFC). Comparison of participants' subjective emotion ratings indicated that all groups showed symmetry between their neural/subjective emotion metrics, and the high-psychopathy group may have showed the greatest such symmetry. These findings suggest that psychopathic individuals may be capable of manifesting emotional reactivity to negatively valent stimuli, at least under certain conditions. Relevance for traditional and developing models of psychopathy is discussed in turn.

## Introduction

Psychopathy exists as a varied constellation of behavioral, emotional and personality-based characteristics that include grandiosity, impulsivity, irresponsibility, shallow affect and a highly parasitic nature (Hare, 2003). These characteristics combine to form a particularly callous and manipulative individual, with poor behavioral controls and a heightened tendency toward antisocial behavior. Indeed, the average incarcerated psychopath has been convicted of five serious crimes by age 40 (Hemphill *et al*., 1998), is substantially more likely to commit violent and recidivistic crimes than non-psychopaths (Hare, 1981; Hare and Hart, 1993) and is less likely to respond effectively to standard therapeutic interventions (Harris and Rice, 2006); but see encouraging recent works by Caldwell and colleagues (Caldwell, 2011; Caldwell *et al*., 2012) and a differing opinion by (Polaschek, 2014).

While the relationship between these varied characteristics has become increasingly delineated (Harpur *et al*., 1988; Hare *et al*., 1990; Neumann *et al*., 2013), the disorder’s underlying etiology remains poorly understood. Historical notions, which remain influential today, posit the psychopath as suffering a chronic hyporesponsivity to aversive and/or negatively valent stimuli (Lykken, 1957; Fowles, 1980; Patrick, 1994; Lykken, 1995; Blair *et al*., 1995; Soderstrom, 2003; Blair, 2005; Patrick, 2007; Rothemund *et al*., 2012; Marsh *et al*., 2013). For instance, the low-fear hypothesis argues that the psychopath may show an inability to muster sufficient fear-related responses to foster effective socialization or to motivate avoidance learning (Lykken, 1957, 1995). A variety of subsequent theories share certain characteristics with this low-fear account. Fowles suggested that psychopaths may be characterized by a hypoactive Behavioral Inhibition System (Fowles, 1980; Fowles and Kochanska, 2000); Blair and colleagues have argued that psychopathic individuals may have a reduced ability to recognize and/or adapt to distress cues in others (Blair *et al*., 1997; Blair, 1999 Blair *et al*., 2004), and other similar hypotheses have been constructed that posit the psychopath as suffering reduced ability to experience and/or process sadness (Blair, 1999; Blair *et al*., 2001; Iria *et al*., 2012), guilt (Blair *et al*., 1995; Johnsson *et al*., 2014), empathy (Soderstrom, 2003; Lishner *et al*., 2015) or negative affect more generally conceived (Hale *et al*., 2004; Seara-Cardoso *et al*., 2016). While differences between these theories may be dissociable and important, they converge in positing the psychopathic individual as lacking a normative capacity to experience, identify and/or process various negative affective states.

Empirical support for these notions, while not universal, has been substantive. Indeed, attenuated emotional experience is perhaps the most widely reported finding in the empirical literature on psychopathy (but see reviews by Lynam and Widiger, 2007; Derefinko, 2015; Hoppenbrouwers *et al*., 2016a for evidence of null, and even positive, associations at times). Subjectively, psychopathic individuals also tend to self-report lower levels of dispositional negative affect (Hicks and Patrick, 2006; Forth and Flight, 2007) as well as reduced subjective reactivity following exposure to emotion-eliciting stimuli (Flor *et al*., 2002; Birbaumer *et al*., 2005; Lishner *et al*., 2015, but see Brook *et al*., 2013). This may be particularly true for moral, mature, emotions (Hoppenbrouwers *et al*., 2016a). And objectively, a broad literature base spanning behavioral, cognitive, physiological and neural research has reported on the psychopathic individual’s attenuated reactivity to negatively valent stimuli. For instance, research has demonstrated reduced identification of negative facial expressions and negative vocal intonations (Blair *et al*., 2002, 2004; Bagley *et al*., 2009; Dawel *et al*., 2012), decreased conditioning to aversive stimuli (Flor *et al*., 2002; Birbaumer *et al*., 2005; Rothemund *et al*., 2012), reduced tendency to pause following aversive feedback (Newman *et al*., 1990), reduced physiological reactivity to impending punishment (Hare *et al*., 1978; Hare, 1982), decreased startle magnitude (Herpertz *et al*., 2001), decreased fear-potentiated startle magnitude (Patrick, 1994; Herpertz *et al*., 2001; Goldin *et al*., 2008; Rothemund *et al*., 2012) and attenuated physiological reactivity when observing others’ distress (Blair *et al*., 1997; Verona *et al*., 2013). Importantly, while some evidence for specific cognitive dysfunction in psychopathy has been reported (Hiatt *et al*., 2004; Hamilton *et al*., 2014; Anderson *et al*., 2015; Hoppenbrouwers *et al*., 2016b; Slotboom *et al*., 2017), and some cognitively-based theories of psychopathy have been posited (e.g. response modulation; Wallace *et al*., 1999; Newman and Baskin-Sommers, 2016) by most accounts, these affective abnormalities do not appear associated with broad cognitive deficits.

Contemporary neuroimaging work has also reported evidence of underlying abnormalities in brain regions believed involved in emotion-related processing, including insula (Birbaumer *et al*., 2005; Decety *et al*., 2013a,b; Meffert *et al*., 2013; Arbuckle and Shane, 2017; Seara-Cardoso *et al*., 2016), amygdala (Yang *et al*., 2009; Ermer *et al*., 2013; Cope *et al*., 2014; Seara-Cardoso *et al*., 2016, but see Müller *et al*., 2003; Schultz *et al*., 2016) and orbitofrontal/ventromedial prefrontal cortex (Soderstrom *et al*., 2002; Ermer *et al*., 2013; Decety *et al*., 2014; Harenski *et al*., 2014). Together these regions form a frontolimbic circuit believed to underlie the detection, interpretation and management of emotional processing (Lindquist *et al*., 2015).

It is important to note, however, that while this broad body of work indicates that the psychopath does not manifest normal responses to emotional stimuli, it does not similarly indicate that they cannot do so*.* For instance, it may be that psychopathic individuals focus less on emotionally relevant stimuli than non-psychopathic individuals (see relevant work by Newman and colleagues; e.g. Wallace *et al*., 1999; Newman and Baskin-Sommers, 2016) or are simply less motivated to manifest an emotional response (Shane and Peterson, 2004; Arbuckle and Shane, 2017). One of the reasons that these distinctions have been difficult to make is because the majority of previous work has tested psychopaths’ emotional processing within what we refer to here as ‘passive-processing’ conditions: conditions within which participants’ naturally occurring reactions are recorded while they serve only as passive recipients of presented stimuli. For instance, in work demonstrating reduced neural responses following images of others in pain (Decety *et al*., 2013a,b; Marsh *et al*., 2013), participants are only asked to watch presented pictures while their naturally occurring psychophysiological/neural reactions are measured. Similarly, even in more complicated paradigms such as fear-potentiated startle (Patrick, 1994; Rothemund *et al*., 2012), participants need only to view presented pictures and listen to startling noise bursts—no active process on the part of the participant is required. While work of this nature has been integral for identifying the naturally attenuated reactivity that characterizes the psychopathic individual, it is insufficient as support for a true ‘inability’ to experience normative levels of emotion.

To approach support for such a core inability, evidence of emotional deficits in situations where the psychopathic individual is ‘trying’ to experience the emotions is necessary. There is a remarkable dearth of such research. Two preliminary studies attempted to assess physiological responses while individuals with differing levels of psychopathic traits viewed stimuli intended to evoke positive and negative emotions. In both of these studies, individuals with varying levels of psychopathic traits were told to ‘watch’ or regulate (either ‘experience’ or ‘suppress’) their emotions towards the emotionally laden stimuli. In one study, no physiological differences were found between individuals scoring high or low in psychopathy (Nentjes *et al*., 2016). The other reported vague relationships between the affective component of psychopathy and heart rate variability (Casey *et al*., 2013).

More recent works by Meffert *et al*. (2013) and Arbuckle and Shane (2017) have utilized MRI to evaluate participants’ neural responses while trying to maximize or minimize their empathy for people in pain. Specifically, participants were asked to either watch the pictures as they normally would (a passive-processing condition) or to try to increase (or decrease) their level of concern for the people in pain (an instructed-processing condition). In both studies, while high-psychopathic participants showed attenuated neural responses within regions comprising a limbic-prefrontal emotion-relevant circuit (i.e. insula, anterior cingulate cortex, IFG, prefrontal cortex) under passive processing, these attenuations were significantly reduced or completely nullified in the instructed-processing condition. Thus, while these studies replicated long-standing empathic deficits during passive processing, they also suggest that individuals high in psychopathy may be capable of normative empathic processing, at least within certain (deliberately conceived) conditions.

These recent studies carry with them several important limitations. First, in both studies the only emotion queried was empathy. Thus, the extent to which psychopathic individuals are equally capable of deliberately manifesting other negative emotions (e.g. fear, guilt) remains unknown. Second, since empathy is a social emotion involving the ability to share another’s affective state (Davis, 1983; Batson *et al*., 1987; Gerdes *et al*., 2010; Bloom, 2017), it is possible that participants in the instructed-processing condition were not actually increasing their own emotional reactions but were instead finding a way to mirror the other person’s emotions. Given that such mirroring has been shown to rely on both affective and cognitive processes (De Vignemont and Singer, 2006; Schnell *et al*., 2011; Walter, 2012; Bloom, 2017), it is difficult to be certain that the high-psychopathic individuals in previous studies were not bolstering their cognitive appreciation for the other’s pain, rather than manifesting a true affective response.

The present study was designed to further research in this area, and to address these two important questions. To this end, probationers/parolees diagnosed for psychopathy via the PCL-R (Hare, 2003) were asked to perform an fMRI-based emotion regulation paradigm during which they viewed a wide variety of negatively valent and neutral pictures from the ‘International Affective Picture System’ (IAPS) database (Lang, 2005). In the ‘Watch’ condition, participants were asked to observe the pictures as they normally would. In the ‘Increase’ and ‘Decrease’ conditions, participants were asked to try to maximize or minimize whatever emotion the pictures naturally evoked in them, respectively.

Given anticipated baseline emotional deficits during passive processing, we hypothesized that psychopathic individuals would show reduced activity compared to non-psychopathic individuals on Neg_WATCH_*vs* Neut_WATCH_ trials within regions underlying emotion-related processing, including bilateral insular, orbitofrontal and inferior frontal cortices. Our primary hypothesis, however, was that psychopathic individuals would show evidence of increased reactivity within these same regions when instructed to voluntarily enhance their emotional response to the negative pictures. This hypothesis could be operationalized through both within- and between-group predictions: (i) within-group: psychopathic (and non-psychopathic) groups should show increased activity within structures comprising the limbic-prefrontal emotion circuit on Neg_INCREASE_ trials compared to Neg_WATCH_ trials and (ii) between-group: psychopathic individuals’ reactivity on Neg_INCREASE_ trials should not differ significantly from the non-psychopathic group’s level of activity on Neg_WATCH_ trials. If this latter hypothesis proved true, it would indicate that the psychopathic group’s level of neural activity under instructed processing equated to the non-psychopathic individuals under passive processing.

## Method

### Participants

Eighty-five individuals (71 males) with criminal records were consented into the study through posted/online advertisements and face-to-face recruitment efforts at a variety of locations throughout the Greater Albuquerque area, including probation/parole offices, halfway houses, drug court, and drug treatment centers. Participant screening was intensive and involved a two-stage process. Initial screening was undertaken over the phone, or via an online questionnaire, and ensured that participants were (i) on probation/parole, (ii) between the ages of 18 and 55, (iii) had a felony-level conviction history, (iv) did not self-report any psychotic disorders in self or first-degree relatives, (v) did not self-report use of any anti-psychotic medication, and (vi) had no contraindicators of MRI (e.g. epilepsy, pregnancy, exposure to metal). Participants passing initial screening were invited to the laboratory for more detailed screening, which included screening for psychiatric disorders (via the Structured Clinical Interview for Axis I and Axis II disorders, SCID; First *et al*., 1997) and for full-scale IQ estimates above 70 (via the two-subtest Wechsler Abbreviated Scale of Intelligence, WASI; Wechsler, 1999). Participants who continued to meet all inclusion criteria following secondary screening proceeded to complete the forensic, MRI and questionnaire assessments, which generally occurred over the course of two separate visits to The Mind Research Network.

Of the 85 consented participants, 6 did not pass secondary screening criteria. In addition, 12 participants were removed from final analyses for the following reasons: 2 had bad MR masks that reduced visualization of key brain regions, 2 experienced computer malfunctions that precluded full data collection and 8 experienced artifacts in their MRI scans due to poor implementation of a new MRI-compatible EEG cap. Thus, data from 67 participants are included in all analyses reported below.

### Clinical and forensic assessments

#### Psychopathy

All participants were assessed for degree of psychopathy via the PCL-R (Hare, 2003), a semi-structured interview that provides in-depth access to relevant details concerning school, family, work and criminal history as well as characteristics of interpersonal and emotional integrity. The assessment is comprised of 20 items (e.g. callous/lack of empathy, superficial charm, poor behavioral control), each of which can be scored 0, 1 or 2. Participants thus obtain a total PCL-R score out of 40; 30 is used as the common cut-off for clinical psychopathy (Hare, 1991). Highly trained research personnel (trained by M.S.S.) conducted each interview and subsequently assigned each participant a score out of 40.

PCL-R scores were assigned based on the comprehensive PCL-R interview alone. Several previous studies have used interview-only techniques to gather informative results (see Harpur *et al*., 1994; Forth *et al*., 1996; Kosson *et al*., 1997). Inter-rater reliability scores are not available; however, all interviews were videotaped for posterity. Mean PCL-R score across all participants was comparable to reports from prison samples (*M* = 22.24; s.d. = 7.42; see Table 1 for complete demographic details). Participants receiving PCL-R scores of 20 or below (n = 23) were classified as `low psychopathy'; participants receiving PCL-R scores between 21-29 (n = 29) were classified as `mid psychopathy'; participants receiving PCL-R scores of 30 or above (n = 15) were classified as `high psychopathy'.

**Table 1:** Demographic and clinical characteristics of the sample

	**Low PCL-R group**				**Mid PCL-R group**				**High PCL-R group**				**Effect sizes**			
	***M***	**s.d.**	***n***	***%***	***M***	**s.d.**	***n***	***%***	***M***	**s.d.**	***n***	***%***	***X*** ^***2***^	***F***	***P***
Gender (% male)			23	87			29	75.9			15	93.3	2.49		*ns*
Age	31.48	10.28			35.38	7.78			33.47	9.45				1.19	*ns*
PCL-R total	14.16	4.12			23.62	2.98			31.98	1.56				145.40	<0.001
Factor 1	4.52	1.73			8.33	2.21			12.40	1.92				71.98	<0.001
Factor 2	8.66	3.35			13.80	2.66			17.07	1.03				48.53	<0.001
Composite drug score	14.91	12.15			28.93	17.10			27.20	18.03				5.51	0.006
Alcohol use over threshold	4.70	8.48			5.41	6.65			7.47	9.37				0.57	*ns*
*SCID diagnoses*															
Psychotic disorders			0	0			0	0			2	13.3	7.15		0.03
Mood disorders			12	52.2			16	55.2			8	53.3	0.05		*ns*
Substance Use Disorders			21	91.3			29	100			15	100	3.94		*ns*

Note: **Alcohol use over threshold** was calculated as the total number of years that an individual consumed five or more drinks of alcohol three or more times per week; **Composite drug score** was calculated as the total number of years an individual regularly used any of the following: heroin, crack/cocaine, methamphetamines, opioids, other amphetamines, methadone, cannabis, hallucinogens or inhalants.

**Fig. 1:**
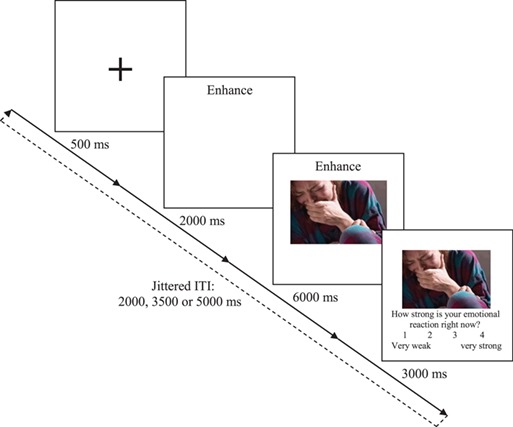
A graphical depiction of the emotion regulation task. Participants received instructions to either *Increase*, *Decrease* or *Watch*, prior to being presented with emotional or neutral images. On *Watch* trials, participants were instructed to allow their emotions to occur naturally. On *Increase* and *Decrease* trials, participants were instructed to try to modulate their emotional responses to the images in the direction indicated.

#### SCID I/P

Participants completed the SCID I/P (First *et al*., 1997), a semi-structured interview that provides diagnostic information regarding major DSM disorders. Highly trained Master’s level research personnel conducted each interview, under the guidance of a senior SCID trainer (R.C.; see Acknowledgements). SCID assessments were used to screen out for specific disorders (see screening criteria above), and to characterize participants’ psychological histories, including antisocial personality disorder and substance abuse disorders.

#### Addiction Severity Index

A modified version of the Addiction Severity Index - Expanded (ASI-X; Segraeus *et al*., 2009) was used to provide a detailed account of each participant’s drug-use history. The ASI-X was administered orally by a trained examiner and indicated the age of first use, the date of last use, and the number of years of regular use, for 14 different substances. The information collected exceed recent PHenX recommendations (Hamilton *et al*., 2011) and afford detailed, psychometrically valuable indices of drug-use severity. Using this, composite drug scores were created by summing the number of years that participants used (i) all drugs, (ii) minor drugs (cannabis, hallucinogens, and inhalants), (iii) major drugs (heroin, crack/cocaine, methamphetamines, opioids, other amphetamines and methadone) and (d) alcohol (Table 1). Total drug-use composite scores were included as covariates in exploratory analyses, to evaluate the robustness of PCL-R-based findings.

#### Weschler Abbreviated Scale of Intelligence (WASI)

Full-scale IQ estimates were obtained via the two-subset WASI (Wechsler, 1999). All participants scored >70 full-scale IQ.

### Emotion regulation task

The emotion regulation task was designed in E-Prime 2.0 (Schneider *et al*., 2002) as a modified version of a well-validated task utilized in previous normative work (Ochsner *et al*., 2002, 2004; Figure 1). Participants viewed a sequence of 72 picture stimuli (54 negatively valent and 18 neutral) and were asked to increase, decrease or maintain their emotional response to the displayed picture. Each trial began with a centered fixation cross, displayed for 1000 ms. After fixation offset, a visual instruction (either ‘INCREASE’, ‘DECREASE’ or ‘WATCH’) was presented for 2000 ms. The picture was then presented for 6000 ms. After picture presentation, participants had 3000 ms to rate their level of subjective emotional reactivity on a 4-point scale from 1 to 4; following a jittered intertrial interval of 2000, 3500 or 5000 ms, the next trial began (Figure 1).

On WATCH trials, participants were instructed to simply watch the pictures naturally. On INCREASE (or DECREASE) trials, participants were instructed to try to maximize (or minimize) the intensity of their naturally occurring emotional response to the picture. The primary focus of the present manuscript surrounded the extent to which psychopaths could increase their emotional reactivity to the aversive stimuli; however, the decrease condition was included as an important control for regulatory ability, and to afford speculative consideration of the influence of psychopathy on the ability to downregulate emotional responses.

Participants performed five practice trials to become familiar with the task. Crucially, participants were not provided with any instructions regarding how to successfully regulate their emotional reactions, as this initial foray into emotional modulation in psychopathic individuals sought to answer the basic question: ‘can psychopaths increase their emotional reactivity, in any manner whatsoever?’ Subsequent work will be required to evaluate the specific strategies utilized, and the extent to which different strategies may elicit different results.

### Picture stimuli

Three sets of 18 negatively valent picture stimuli (1 for each of the Neg_WATCH_, Neg_INCREASE_ and Neg_DECREASE_ conditions) and 1 set of 18 neutral picture stimuli (for the Neut_WATCH_ condition) were used by selecting pictures from the IAPS database (Lang, 2005) and supplementing additional images from the internet. The negatively valent picture sets included images of spiders, snakes, dead bodies and people crying; the neutral picture sets included images of household items, buildings and people talking, typing or working. Each of the three negatively valent picture sets was matched carefully for content, such that each set included a picture of a snake, a picture of a spider, a picture of a burn victim, a picture of a person crying, and so on. Additionally, picture sets were paired with each instruction condition in counterbalanced fashion, such that a given picture set was matched with a given instruction condition an equal number of times across all participants. Thus, any differences in participants’ reactivity profiles could not be the result of differences in picture content.

### Data aquisition

All fMRI data collection was performed using a Siemens TIM Trio 3 Tesla MRI system. Images were presented with a JVC DLA Multimedia projector (Model DLA-SX200-NLG) using E-Prime 2.0 software (Schneider *et al*., 2002). Thirty-three axial slices (3.5 mm) covering the whole brain were collected using a gradient echo-planar pulse sequence (TR, 2000 ms; TE, 29 ms; FOV, 24 × 24 cm, 64 × 64 matrix; voxel size, 3.8 mm × 3.8 mm × 3.5 mm; flip angle, 75°). Functional images were reconstructed offline and reoriented to approximately the anterior commissure/posterior commissure plane. Functional image runs were motion corrected using an algorithm unbiased by local signal changes (INRIAlign; Freire *et al*., 2002). Motion spikes > 4 mm were nearest-neighbor replaced via the artifact repair tool (ARTRepair; Mazaika *et al*., 2009)*.* A mean functional image volume was constructed for each run from the realigned image volumes. The mean echo-planar image (EPI) image was normalized to the EPI template. The spatial transformation into standard Montreal Neurological Institute space was determined using a tailored algorithm with both linear and non-linear components (Friston *et al*., 1995). The normalization parameters determined for the mean functional volume were then applied to the corresponding functional image volumes for each participant. The normalized functional images were smoothed with a 9 mm full width at half-maximum Gaussian filter. A high-pass filter (cut-off period 116 hz) was applied to remove any low-frequency confounds.

### Data analysis

Individual participant data was analyzed using a mixed-effects event-related model in Structural Parameter Mapping 12 (SPM12). Instruction (2 s duration), picture (6 s duration) and rating (3 s duration) were all modeled as separate events, with a standard hemodynamic response function. Picture served as the primary event of interest, with contrast images created separately for Neg_WATCH_, Neg_INCREASE_, Neg_DECREASE_ and Neut_WATCH_ conditions. Events were entered into a random-effects ‘flexible factorial’ model in SPM12 to create a 3 (Group: high-psychopathy, mid-psychopathy, low-psychopathy) × 4 (Trial Type: Neut_WATCH_, Neg_WATCH_, Neg_INCREASE_, Neg_DECREASE_) repeated-measures analysis of variance (ANOVA), with a within-group ‘Subject’ factor included in the model. Evaluation of higher-order main effects and interactions were followed by t-contrasts, guided by a priori hypotheses, which focused on the [Neg_WATCH_*vs* Neut_WATCH_] and [Neg_INCREASE_*vs* Neg_WATCH_] contrasts (to evaluate reactivity under passive processing and instructed-processing conditions, respectively). Neg_DECREASE_ conditions were also evaluated but had no specific hypotheses attached to them.

At the request of insightful peer reviewers, additional analytic models were created to evaluate the stability of reported effects with important covariates included in the model: age, gender, SCID diagnosis and composite substance use score. These covariate analyses were undertaken within the Multivariate and Repeated-Measures (MRM) toolbox (Mcfarquhar *et al*., 2016) because the influence of subject-level covariates will necessarily be zero within any SPM ‘flexible factorial’ model with subject factor included. Analyses reported within are those undertaken within the original SPM model, as all reported findings were only minimally influenced by the inclusion of covariates. All raw data, and the MRM-based covariate analyses, are available upon request.

All data were intensity-thresholded at *P* < 0.001, with a cluster size correction (*k* = 29) undertaken via RESTPlus AlphaSim (Song *et al*., 2011) using a FWHM of 9, rmm of 5, and 1000 iterations, to equate to a family wise error (FWE) rate of *P* < 0.05. In addition, five 10 mm regions of interest (ROI) spheres were obtained from a recent meta-analysis on emotion processing by Lindquist *et al*. (2015) and constructed within bilateral insula (−39, 24, −12; 42, 24, −9), bilateral amygdala (24, 3, −18; −27, −6, −18) and ventromedial prefrontal cortex (−3, 39, 0). All ROI analyses were thresholded at *P* < 0.05, FWE-svc.

## Results

### Psychopathy scores and demographic data

All psychopathy and demographic data are displayed in Table 1. As expected, a one-way ANOVA confirmed that the three groups differed significantly on PCL-R total score, *F*(2,64) = 145.40, *P* < 0.001 as well as Factor 1, *F*(2,64) = 71.98, *P* < 0.001 and Factor 2, *F*(2,62) = 48.53, *P >* 0.001, scores. Additionally, analyses were conducted to determine if there were group differences in age, gender, alcohol/substance use and comorbid DSM disorders. A group difference in substance dependence was identified, χ^2^ = 9.11, $P < 0.01$; however, all other demographic/clinical/forensic variables showed no differences across groups.

**Fig. 2:**
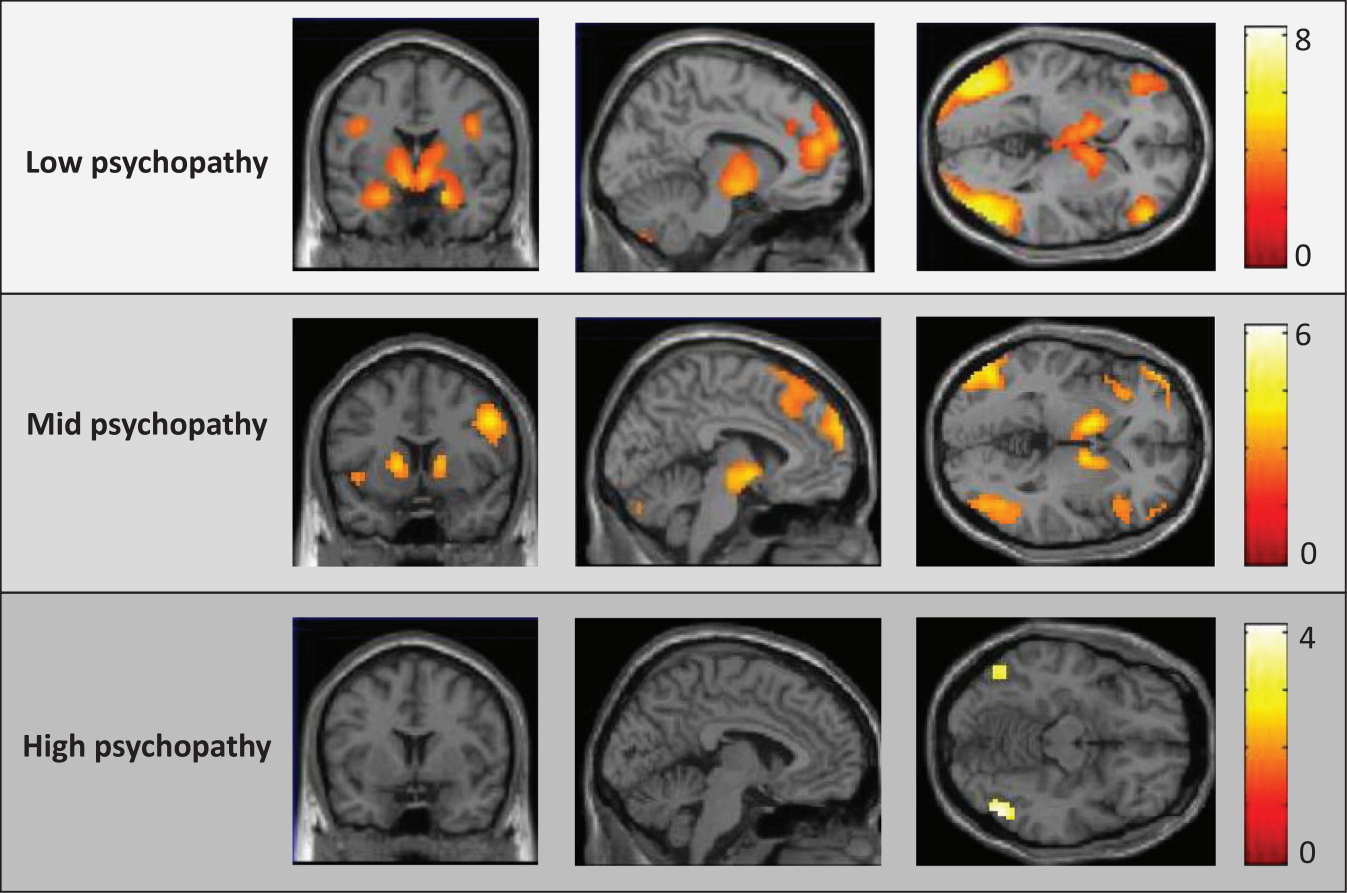
Neural activity within the Neg_WATCH_ > Neut_WATCH_ contrast for each of the low-, mid- and high-psychopathy groups. SPM thresholded at $P < 0.005$, uncorr. for display purposes.

**Fig. 3:**
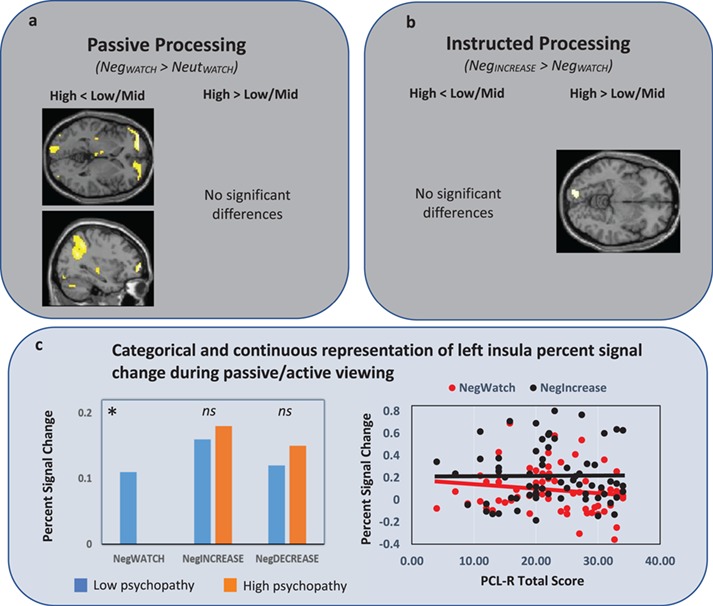
Whole-brain group differences in neural response under passive (3a) and instructed (3b) processing conditions. Note the high psychopathy group showed reduced activity during passive, but not instructed, processing within ventromedial/ventrolateral PFC and angular gyrus. ROI analyses (3c) identified a similar reduction during passive, but not instructed, processing within left insula.

### Subjective ratings of emotional reactivity

A 3 (Group: High, Mid, Low Psychopathy) × 4 (Trial Type: Neut_WATCH_, Neg_WATCH_, Neg_INCREASE_, Neg_DECREASE_) mixed-model ANOVA revealed a significant main effect of TrialType, *F* = 99.08, *η^2^* = 0.61, *P* < 0.001. As expected, emotion ratings were lower on Neut_WATCH_ trials, *M =* 1.49 (0.53), compared to each of the Neg-picture trials (all *P*s < 0.01). Within the Neg-picture trials, emotion ratings were higher on Neg_INCREASE_, *M = 2.77 (0.51)* than Neg_WATCH_ trials, *M = 2.39 (0.56)*, *P* < 0.05, which were in turn higher than on Neg_DECREASE_ trials, *M = 2.10 (0.59), P* < 0.05. The main effect of Group and the Group × TrialType interaction did not reach significance (*P*s > 0.05, respectively).

### Neural indicators of emotional reactivity

#### Higher-order effects

A main effect of TrialType exhibited widespread activity across the entire brain [*k* = 22664; *peak F* (left parahippocampal cortex) = 50.12], with bilateral subpeaks developing in several regions including orbitofrontal/insular cortex, inferior temporal cortex, precuneus, dorsal anterior cingulate and supplementary motor regions (Supplementary Figure S1). Subsequent comparisons indicated that activity was lower on Neut_WATCH_ trials compared to all three Neg-picture trial types (Supplementary Table S1) and lower on Neg_WATCH_ trials compared to both Neg_INCREASE_ and Neg_DECREASE_ trials (Supplementary Table S2 and S3). The main effect of Group also exhibited widespread activity [*k* = 41701, *peak F* (right parahippocampal) = 164.34], with bilateral subpeaks arising within several regions, including angular, postcentral, orbitofrontal/insular, midfrontal and occipital regions (Supplementary Figure S2). Subsequent comparisons indicated that low-psychopathy participants exhibited overall greater activity than mid-psychopathy participants, who in turn exhibited overall greater activity than high-psychopathy participants (Supplementary Table S4–6). These differences were widespread but were particularly prominent within superior parietal, inferior frontal, temporal, orbitofrontal, insular and cingulate cortices*.* These main effects were influenced by a significant Group × TrialType interaction within the left insula ROI (Figure 3C).

#### Reactivity to emotional pictures under passive viewing

The Neg_WATCH_ > Neut_WATCH_ contrast provided a well-controlled evaluation of participants’ baseline reactivity to the emotionally valent pictures. Across the entire sample, this contrast revealed robust activity, with notable peaks within left dorsomedial prefrontal cortex, bilateral insular/inferior frontal cortices, bilateral amygdala, bilateral thalamus/nucleus accumbens and bilateral occipital cortices. Evidence of deactivation was equally prevalent, with notable deactivation peaks within bilateral fusiform/lingual/precuneus, left superior temporal cortex spreading into temporoparietal junction (TPJ) and right postcentral cortex (see Supplementary Table S7 and Supplementary Figure S4 for all significantly activated and deactivated regions).

**Fig. 4:**
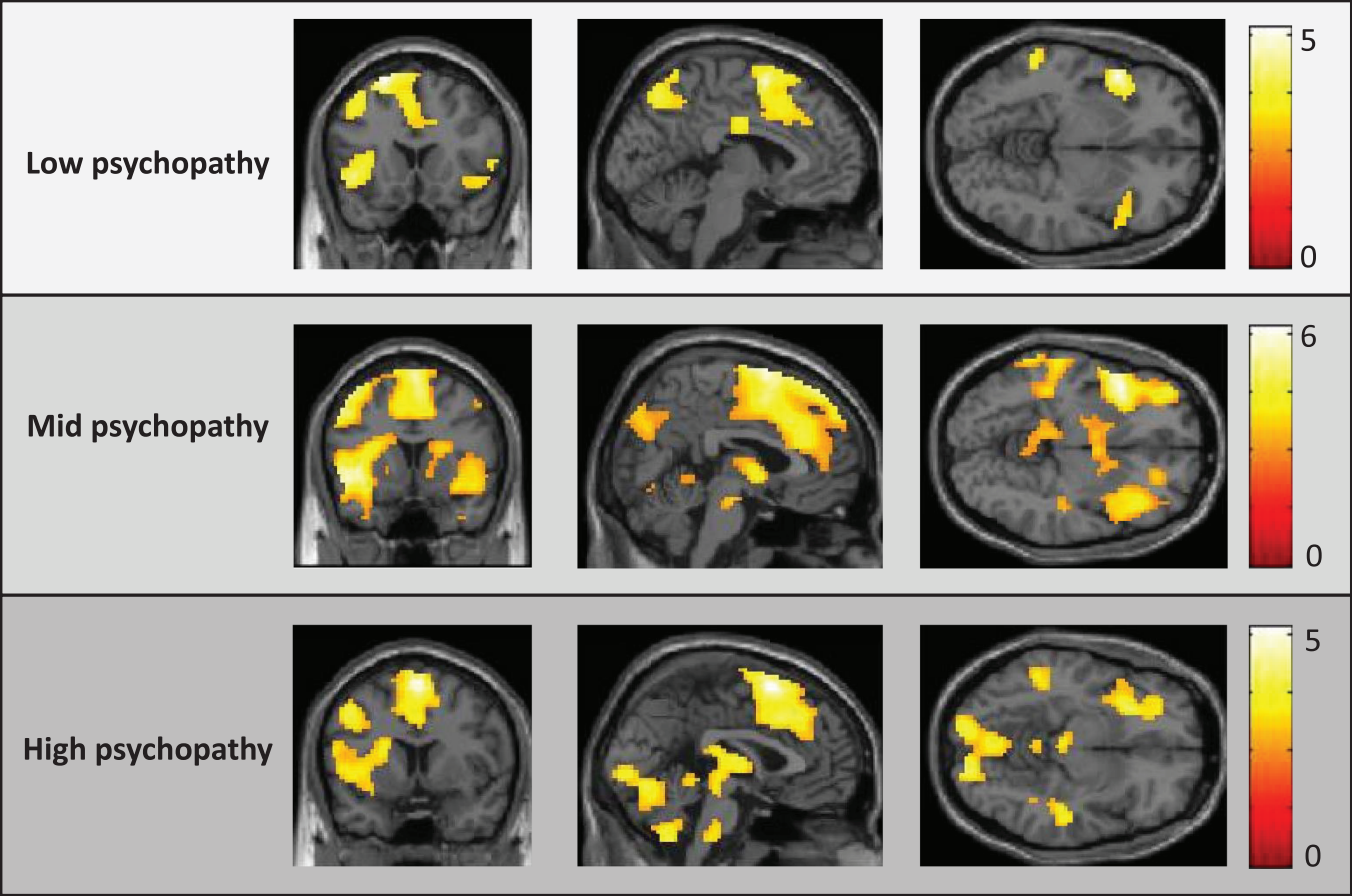
Neural activity within the Neg_INCREASE_ Neg_WATCH_ contrast for each of the low-, mid- and high-psychopathy groups. SPM thresholded at $P < 0.005$, uncorr. for display purposes.

Group-specific activity was first evaluated via one-sample *t*-tests, followed by direct comparison of high-, mid- and low-psychopathy groups. One-sample *t*-tests indicated that both the low- and mid-psychopathy groups showed significant bilateral activity throughout amygdala, thalamus/lentiform, orbitofrontal/insular, inferior frontal, dorsomedial and occipital cortices. In contrast, the high-psychopathy group showed only small regions of activity within right amygdala and bilateral occipital cortex (Figure 2; Supplementary Table S8–10). Group comparisons indicated that the low and mid psychopathy groups showed no regions of differential response; however, the high-psychopathy group showed reduced response compared to the low-/mid-psychopathy groups within bilateral ventromedial/ventrolateral, left angular and left occipital cortices (Figure 3A; Supplementary Table S11). Of note, group differences were not found in the amygdala—one of the few regions where the high-psychopathy group showed activity in response to the negative pictures (see other instances of this: no differential amygdala activation in response to processing positive pictures, Müller *et al*., 2003; in processing emotional facial expressions, Decety *et al*., 2014). No regions showed increased activity in the high-psychopathy group.

#### Reactivity to emotional pictures under instructed upregulation

The Neg_INCREASE_ > Neg_WATCH_ contrast provided a well-controlled evaluation of change in participants’ reactivity to the emotionally valent pictures when instructed to maximize their emotional response to the images. Preliminary analyses across the entire sample indicated that participants were able to undertake this instructed upregulation; robust increases in neural response were seen across frontal, parietal, temporal and occipital regions [*k* = 14225, *peak t* (supplementary motor area) = 7.50] with notable peaks within dACC/mFC, bilateral insular/orbitofrontal cortices, bilateral amygdala, right middle frontal and right inferior parietal cortices (Supplementary Figure S5 and Supplementary Table S2). No regions showed decreased activity in the Neg_INCREASE_ condition.

Group-specific activity was first evaluated via one-sample *t*-tests, followed by direct comparison of high-, mid- and low-psychopathy groups. One-sample *t*-tests indicated that all three groups showed robust increases in neural response throughout several regions including left insular/orbitofrontal cortices, anterior cingulate/medial frontal cortices and inferior parietal regions (Figure 4; Supplementary Table S12–14). Direct group comparisons identified no regions where the high-psychopathy group showed reduced activity compared to the low-/mid-psychopathy groups (Figure 3B). However, the high-psychopathy group showed increased activity in right occipital cortex compared to the low-/mid-psychopathy groups (Supplementary Table S15).

#### PSC in ROI regions

The above whole-brain analyses suggest that the emotion-related attenuations seen in the high-psychopathy group under passive processing may have been reduced under instructed processing. To test this more formally, we calculated PSC values within our a priori ROIs and undertook both categorical (between-group *t*-tests) and continuous (regression) analyses to evaluate variation in PSC versus the Neut_WATCH_ condition. Between-group *t*-tests indicated that within the left insula ROI, the high-psychopathy group showed reduced PSC in the Neg_WATCH_ condition, but not in the Neg_INCREASE_ condition. Regression analyses further characterized these effects: across the entire sample, left insula PSC values in the Neg_WATCH_ condition correlated negatively with PCL-R total scores (Figure 3C), but no association was found in the Neg_INCREASE_ condition. Importantly, these negative relationships held after controlling for composite drug-use scores, SCID dependence diagnoses and IQ.

Finally, between-group *t*-tests on the high-psychopathy group’s Neg_INCREASE_ PSCs and the low-/mid-psychopathy groups’ Neg_WATCH_ PSCs identified no regions with significant difference, even using a liberal significance threshold of uncorrected *P* < 0.05. Thus, the high-psychopathy group’s neural response in the Neg_INCREASE_ condition was of a magnitude that was undifferentiable from the low-psychopathy group’s neural response under passive-processing conditions.

#### Reactivity to emotional pictures during decrease instruction

The Neg_WATCH_ > Neg_DECREASE_ contrast was somewhat tangential to primary study aims but nonetheless afforded a useful evaluation of the relationship between psychopathy and the ability to downregulate emotional responses. Participants appeared to have a difficult time with this task overall, as no regions showed significant reductions in the Neg_DECREASE_ condition. Rather, we observed increased responses within several key regions, including left inferior parietal, bilateral insular, right mid-temporal, right orbitofrontal and bilateral cingulate/midfrontal cortices (Figure S6 and Supplementary Table S3). Difficulty with downregulation is something we have seen in previous work (Shane and Weywadt, 2014). No group differences in downregulation ability were identified via one-way ANOVA or in targeted group contrasts (high- psychopathy *vs* low-psychopathy; high-psychopathy *vs* mid-psychopathy), suggesting that the high-psychopathy group showed no noticeable deficits in downregulation ability.

#### Increase *vs* decrease instructions

While increased neural responses in the ‘Increase’ condition could represent increased emotional expression, it could also represent increased cognitive effort required to undertake purposeful upregulation. To evaluate the plausibility of this alternate hypothesis, we directly contrasted the Neg_INCREASE_ and Neg_DECREASE_ conditions because the similar regulatory demands of these two conditions should control for activity associated with regulatory effort. This contrast identified increased activity in the Neg_INCREASE_ condition within several regions including left insula/amygdala, thalamus/caudate, anterior cingulate and bilateral occipital cortex (Supplementary Figure S7 and Supplementary Table S16). Only one cluster within right angular cortex displayed significantly reduced activity in the Neg_INCREASE_ condition.

Interestingly, one-sample *t*-tests indicated that only the mid- and high-psychopathy groups emulated this pattern of activity; the low-psychopathy group showed increased relative Neg_INCREASE_ activity only within bilateral occipital cortex, and showed decreased relative Neg_INCREASE_ activity within right dorsomedial and dorsolateral, right inferior parietal and right orbitofrontal cortices. Between-group *t*-tests were consistent with these findings: no differences were found between the high- and mid-psychopathy groups; however both groups showed increased relative Neg_INCREASE_ activity in several ROI regions compared to the low-psychopathy group (Supplementary Table S17 and 18). One interpretation of this is that the mid- and high-psychopathy groups may have evidenced a greater range between their maximum (Neg_INCREASE_) and minimum (Neg_DECREASE_) neural responses.

### Relationship between neural and subjective levels of emotional reactivity

**Fig. 5:**
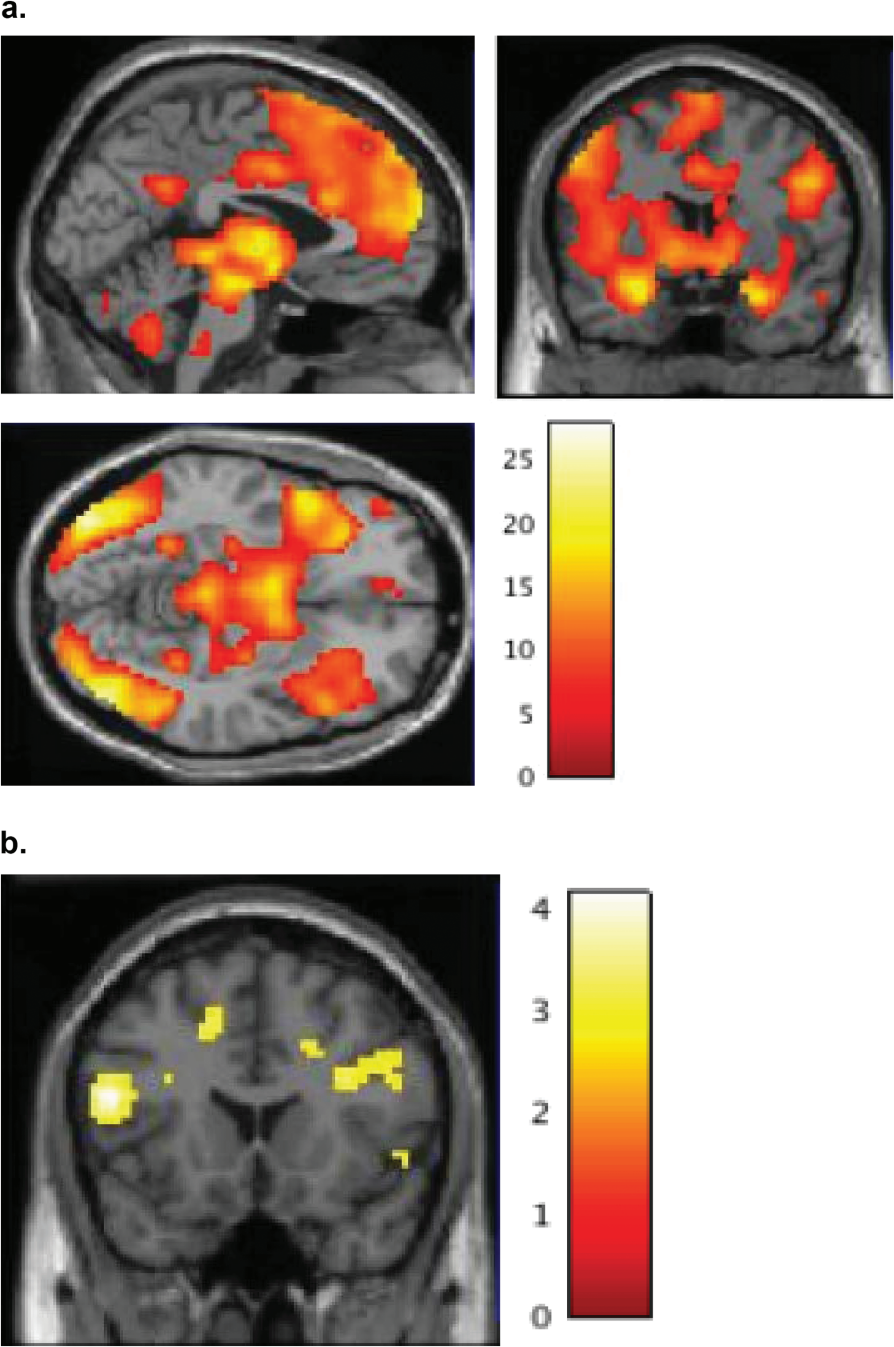
(a) Brain regions where activity varied parametrically with subjective emotion ratings on a trial-by-trial basis (ie. trial-by-trial neural/subjective synchrony). (b) Brain regions where the magnitude of neural/subjective synchrony correlated positively with PCL-R scores (no negative correlations were found).

Finally, we sought to evaluate the relationship between participants’ neural reactivity to the presented pictures and their subjective ratings of emotional experience. To this end, we undertook a parametric modulation analysis, using participants’ subjective emotion ratings as a trial-by-trial modulator of neural response within a one-way ANOVA. This whole-brain analysis identified regions where the magnitude of activity correlated with participants’ subjective ratings. Consistent with hypotheses, across all participants we found robust positive relationships within two large clusters (*k* = 4293 and *k* = 11071) with subpeaks in bilateral OFC/IFG/insula, bilateral ACC/mFC/SMA and bilateral limbic centers (Figure 5A; Supplementary Table S19). No regions showed a negative relationship with subjective emotion ratings*.*

To evaluate potential differences between neural/subjective symmetry and psychopathy, we conducted a correlational analysis between this parametrically modulated data and participants’ psychopathy scores. This analysis identified a positive correlation—indicative of a ‘higher’ neural/subjective symmetry—in high-psychopathy scorers within several regions, including bilateral IFG, left TPJ and left inferior parietal cortex (Figure 5B; Supplementary Table S20). No regions were identified that showed a negative correlation with psychopathy scores. These correlational analyses remained significant after controlling for IQ and total substance use. Moreover, they were confirmed by one-sample *t*-tests, which indicated that all three psychopathy groups showed significant neural/subjective symmetry within regions including bilateral insula, bilateral thalamus/caudate and bilateral dACC/mFC (Supplementary Figure S8), and between-group *t*-tests that indicated that the high-psychopathy group showed greater symmetry than the low-psychopathy group within these regions (Supplementary Figure S9).

## Discussion

The present study sought to evaluate the extent to which psychopathic individuals would show evidence of neural reactivity to negatively valent stimuli under either passive- or instructed-processing contexts. To this end, high-psychopathy participants (>30 on the PCL-R) showed significantly attenuated neural responses to negatively valent pictorial stimuli under passive-processing conditions, which were significantly reduced when they were instructed to try to maximize (and potentially also when instructed to minimize) their naturally occurring emotional reactions to these same pictures. The locations of these increased neural responses included several regions involved in the generation of basic emotional responses (Lindquist *et al*., 2015), and which have often been shown to be attenuated in psychopathic populations (e.g. Hastings *et al*., 2008; Decety *et al*., 2013a; Seara-Cardoso *et al*., 2016). Thus, despite baseline attenuations, high-psychopathy participants appeared capable of deliberately manifesting emotional responses to the negatively valent pictorial stimuli within several regions believed to underlie emotional processing. Of additional note, the magnitude of these deliberately evoked emotional responses was comparable to levels exhibited by low-psychopathy participants’ during passive processing. Thus, high-psychopathy participants not only increased their neural sensitivity to the negative pictures in amounts equal to or greater than the low-psychopathy participants, but these increases brought their absolute level of Neg_INCREASE_ activity to levels that suggested some ‘normalization’ of these responses.

While replicating the extant literature, which has long established the psychopaths’ hyporesponsivity to aversive stimuli (Lykken, 1957, 1995; Fowles, 1980; Blair, 2005; Patrick, 2007), these findings also dovetail with recent work from our laboratory (Arbuckle and Shane, 2017) and others (Meffert *et al*., 2013) that has shown an ability for high-psychopathy individuals to deliberately manifest vicarious responses for others. Incapacity models of psychopathy should hypothesize an inability to undertake such deliberate upregulation of emotion, given that the psychopath is deemed to have broadly decreased access to, and experience from, their emotional system. However, in the present study high-psychopathy participants were capable of increasing their neural responses within emotion-generating regions when instructed to try to maximize those responses (and showed the greatest Neg_INCREASE_-to-Neg_DECREASE_ differential within these regions). Of particular note, the high-psychopathy participants also showed equal subjective ratings of emotional reactivity and greater symmetry between their neural and subjective ratings than the low-/mid-psychopathy groups. This congruence is consistent with the notion that the high-psychopathy participants’ deliberately-evoked neural activity may have in fact led to a similar increase in their subjective emotional states.

Whereas Meffert *et al*. (2013) and Arbuckle and Shane (2017) focused on the ability to deliberately increase one’s consideration for another’s experiences, the present study asked participants to increase their ‘own’ naturally occurring emotional reactions to a wide variety of negatively valent pictures. This minor methodological change may have at least three important implications. First, by presenting stimuli depicting a wide variety of negative emotions, including fear, sadness and disgust, we demonstrate that the ability for high-psychopathy individuals to intentionally invoke emotional responses above and beyond their natural baseline state may be a more general capacity that extends past concern for another’s experiences. Second, as participants in our study were asked to experience and modulate their own emotions, rather than to try to feel for another person, we have increased assurance that changes in neural reactivity represent changes in participants’ own emotional experiences. Third, rather than indicating a specific emotion to experience, task instructions specifically requested that participants attempt to modulate ‘whatever emotion the presented pictures naturally invoke in you’. This instruction was devised carefully, to ensure that the modulated emotion (i) was the same one experienced in the Neg_WATCH_ condition and (ii) would only be modulated if the picture did indeed naturally evoke some emotion in the participant. Thus, these results help show that psychopathic individuals are capable of having ‘normative’ emotional experiences.

Finally, by including both Neg_INCREASE_ and Neg_DECREASE_ conditions, the present study included conditions within which regions underlying the generation *vs* the regulation of emotion, should show opposite patterns of activation. Thus, by comparing the Neg_INCREASE_ and Neg_DECREASE_ conditions, we held some ability to differentiate between neural responses representing the generation of a true emotional response from neural responses representing the recruitment of emotion regulation processes. Results revealed increased activity within the Neg_INCREASE_ condition within all three groups, suggesting that regardless of psychopathy scores, participants were able to effectively increase their emotional reactions to the pictorial stimuli when instructed to.

In total, the present study demonstrates that psychopathic individuals were capable of manifesting a wide variety of emotional responses when asked to try to do so. Please do not misconstrue; however, we are not trying to suggest that psychopathic individuals do generate or experience appropriate levels of emotion in their day to day lives—indeed, considerable research and substantive anecdotal data clearly demonstrate that they do not. However, there is an important distinction between ‘cannot’ and ‘do not’ and confirmation that psychopaths are capable of such emotional experience—even if only within constrained contexts—may be difficult for incapacity models of the disorder to synthesize.

With this in mind, it is worth noting several etiological theories of the disorder that do not rely on low-fear frameworks (e.g. Eysenck and Eysenck, 1976; Gorenstein and Newman, 1980; Shane and Peterson, 2004). One such theory is Newman and colleagues’ ‘response modulation’ model (Newman *et al*., 1990, 1997). Instead of arguing for a core emotional deficit, this model proposes an underlying attentional abnormality that precludes the psychopath’s ability to allocate sufficient attention to emotional information once they are locked into a goal-directed set. While the present study remains largely agnostic to this model, we acknowledge that the psychopath’s ability to demonstrate emotional processing only when such processing was framed as the primary task is generally consistent with Newman’s response modulation hypothesis.

A second theory, introduced recently by several research groups (Arbuckle and Shane, 2017; Vitale *et al*., 2018), has posited that the psychopath’s reduced emotional experiences may stem from differences in motivation rather than ability. According to these models, the psychopath may be perfectly capable of experiencing emotion when so inclined, but may rarely become sufficiently motivated to do so. Interestingly, motivational accounts dovetail somewhat with the response modulation hypothesis, in that they both predict the psychopath will show capacity for emotional experience when the psychopath allocates dedicated resources toward the task. However, whereas response modulation posits that the lack of dedicated resources within the majority of contexts stems from a subtle cognitive deficit, motivational accounts more straightforwardly suggest the psychopath may prefer to not process emotional—particularly negatively valent—information.

One particular theory, put forward initially by Shane and Peterson (2004), further posited that the psychopath may be particularly adept at utilizing cognitive or affective strategies intended to minimize the impact that processing of aversive information would otherwise entail. According to these authors, a strategic minimizing of negative affect may induce a hyporeactivity to aversive information that could mimic a core inability to experience negative affect. Indeed, an established literature has revealed that healthy individuals are quite capable of such minimization (Gross and Levenson, 1997; Gross, 2002; Ochsner *et al*., 2002; Goldin *et al*., 2008), and that individuals who undertake such strategies can show an apparent insensitivity to aversive information, including reduced fear-potentiated startle (Temple and Cook, 2007), increased pain tolerance (Jamner and Schwartz, 1986), inferior passive avoidance learning (Shane and Peterson, 2004) and reduced neural response to fearful faces (Rauch *et al*., 2007). The overlap between these characteristics and many of the well-established features of psychopathy is circumstantial but intriguing. At least one aspect of the present findings may add additional credibility to the Shane and Peterson (2004) model, however: the psychopathic group in the present study did not simply demonstrate normal voluntary modulation of emotional reactivity, but rather showed greater increases in hemodynamic response on Neg_INCREASE_ trials than did the non-psychopathic group within left insula, bilateral amygdala, bilateral inferior frontal, bilateral hippocampus and right middle frontal cortex. Moreover, while they did not show superior ability to downregulate their emotional responses, they did show greater Neg_INCREASE_-to-Neg_DECREASE_ range. Finally, they also showed greater symmetry between their neural and subjective indicators of emotional reactivity. In total, these findings suggest that psychopathic individuals may be particularly adept at invoking control over their emotional output; however, additional work will be necessary to further investigate this possibility (and to more comprehensively investigate a wider range of emotion regulation strategies, i.e. antecedent- *vs* outcome-focused strategies; Ochsner and Gross, 2005).

There are a number of notable weakness and limitations that should be identified. First, we must note our modest sample size, which highlights a constant challenge in research on psychopathy. Our sample was sufficient to reveal significant effects across our intended analyses; nonetheless, the findings must remain somewhat preliminary until they can be replicated within a larger sample of participants. Second, we would be remiss not to note that our PCL-R assessments were conducted as interview-only assessments, without subsequent file review. While we recognize that this interview-only format limits somewhat a comparison to the extant literature, prior research has demonstrated the utility of interview-only formats (e.g Forth *et al*., 1996; Arbuckle and Shane, 2017). Third, while we report robust activation across the majority of the limbic-prefrontal emotion circuit to the negative pictures, we must note the relative lack of differences between our groups in amygdala activation in the Neg_WATCH_ condition. One possibility is that the broad array of emotions evoked by our picture set inconsistently recruited the amygdala. However, it is also worth noting that amygdala dysfunction in psychopathy may not be as prominent as originally hypothesized, and studies have shown that those with high-psychopathy scores have increased amygdala activation toward emotional images (e.g. Müller *et al*., 2003; Decety *et al*., 2013a). Furthermore, when comparing our high-, mid- and low-psychopathy groups’ neural responses in the Neg_WATCH_ > Neut_WATCH_ contrast, we found consistent amygdala responses within all groups. While we can only speculate on the similar amygdala responses across groups, we note that our picture set was designed to invoke a broad array of negative emotions, including fear, sadness and disgust. Our use of a picture set that invoked a broader range of negative emotions may, then, have more reliably triggered these structures within the limbic-prefrontal circuit.

Despite these limitations, we believe the present findings indicate that psychopathic individuals can show neural activity suggestive of normal levels of emotional reactivity, at least under certain laboratory situations. These findings, if true, may encourage a reconsideration of current models of the disorder that posit core dysfunction in the processing and/or experiencing of negative emotions. To this end, it is important to recognize the implications of incapacity models, which may reach far beyond etiological considerations of the disorder and may have sweeping consequences that span clinical, forensic and judicial concerns. For instance, if psychopaths are deemed incapable of experiencing negative affect at levels that can guide adaptive behavior, then preventative and therapeutic strategies aimed at cultivating increased reactivity will be deemed doomed from the start (indeed, psychopathy is broadly considered untreatable at present, but see Caldwell, 2011; Caldwell *et al*., 2012; Polaschek, 2014; Caldwell *et al*. 2016 for alternate perspectives). Management of psychopaths will be confined to keeping them behind bars (indeed, this remains true today as well). More speculatively even, incapacity models could have implications for legal models of criminal responsibility. While acknowledging that some of these issues span beyond the domains of psychology and neuroscience, we believe it useful—and perhaps motivating—for researchers to recognize the extent to which their research may influence broader aspects of society.

## Supplementary Material

Supplementary Data
