# Supplementary material for: Capacity for upregulation of emotional processing in psychopathy: all you have to do is ask
Source: Soc Cogn Affect Neurosci. 2018 Sep 25;13(11):1163–76. doi: 10.1093/scan/nsy088 (PMC6234320; doi:10.1093/scan/nsy088)
Supplement: Supplementary Data [file nsy088_suppl_data.zip › scan-17-477-File011.docx]

Table s4. Regions showing differential activity between Low Psychopathy and Mid Psychopathy Groups.

| **Region** | **L/R** | **Peak coordinate** | **Cluster size** | **t-score** |
| --- | --- | --- | --- | --- |
| *Low Psychopathy Group > Mid Psychopathy Group* | | | | |
|  |  |  |  |  |
| *SPC/AMY/AI/vmPFC/Parahippocampal* | Bilateral | -24, -36, 60 | 15 054 | 15.19 |
|  |  | 18, -54, 63 |  | 15.18 |
|  |  | 36, -27, -21 |  | 13.95 |
|  |  |  |  |  |
| Brain Stem | Right | 3, -45, -42 | 304 | 9.39 |
|  |  | 9, -51, -51 |  | 7.16 |
|  |  | 9, -27, -24 |  |  |
|  |  |  |  |  |
| Inferior Frontal Cortex | Right | 39, 9, 24 | 58 | 7.36 |
|  |  |  |  |  |
| *Mid Psychopathy Group > Low Psychopathy Group* | | | | |
|  |  |  |  |  |
| Occipital Cortex | Bilateral | 27, -93, 6 | 2427 | 14.57 |
|  |  | -6, 99, 0 |  | 13.55 |
|  |  | -12, -87, -6 |  | 13.49 |
|  |  |  |  |  |
| Middle Frontal Cortex | Left | -33, 51, 30 | 4244 | 12.70 |
|  |  | -24, 42, 48 |  | 12.27 |
|  |  | -30, 45, 42 |  | 12.11 |
|  |  |  |  |  |
| Precuneus |  | 18, -54, 39 | 55 | 7.18 |
|  |  |  |  |  |
| Inferior Temporal Cortex | Left | -36, -42, 0 | 180 | 7.12 |
|  |  | -51, -42, -12 |  | 6.05 |
|  |  | -42, -33, -15 |  | 6.03 |
|  |  |  |  |  |
| Superior Parietal Cortex | Right | 39, -60, 60 | 44 | 7.04 |
|  |  | 45, -66, 51 |  | 4.69 |
|  | Left | -15, -78, 54 | 41 | 5.71 |
|  |  | -24, -75, 54 |  | 3.90 |
|  |  | -12, -63, 39 |  | 3.88 |
|  |  |  |  |  |
| SMA | Left | -9, -6, 54 | 31 | 5.41 |
|  |  |  |  |  |
| Inferior Parietal Cortex | Left | -27, -51, 42 | 29 | 4.73 |
|  |  |  |  |  |

Note: SPC = superior parietal cortex; AMY = amygdala; AI = anterior insula; vmPFC = ventromedial prefrontal cortex; SMA = supplementary motor area

Whole-brain t-scores in this table were cluster-thresholded at p < .001, to equate to p < .05, FWE. Italicized regions indicate whole-brain clusters that overlapped with ROI regions
