# Supplementary material for: Capacity for upregulation of emotional processing in psychopathy: all you have to do is ask
Source: Soc Cogn Affect Neurosci. 2018 Sep 25;13(11):1163–76. doi: 10.1093/scan/nsy088 (PMC6234320; doi:10.1093/scan/nsy088)
Supplement: Supplementary Data [file nsy088_suppl_data.zip › scan-17-477-File013.docx]

Table s6. Regions showing differential activity between Mid Psychopathy and High Psychopathy Groups.

| **Region** | **L/R** | **Peak coordinate** | **Cluster size** | **t-score** |
| --- | --- | --- | --- | --- |
| *Mid Psychopathy Group > High Psychopathy Group* | | | | |
|  |  |  |  |  |
| *ITC/MFC/AI/AMY/vmPFC*/Cuneus | Bilateral | -27, 30, 18 | 26 815 | 14.26 |
|  |  | -39, -33, -15 |  | 14.25 |
|  |  | -6, 96, 3 |  | 13.26 |
|  |  |  |  |  |
| *High PCL-R > Mid PCL-R* |  |  |  |  |
|  |  |  |  |  |
| Medial Frontal Cortex | Bilateral | 15, 48, -3 | 395 | 9.59 |
|  |  | 12, 36, -15 |  | 8.43 |
|  |  | -12, 24, -12 |  | 5.36 |
|  |  | -24, 48,-3 | 43 | 4.67 |
|  |  | -18, 57, 6 |  | 4.53 |
|  |  |  |  |  |
| Superior Frontal Cortex | Right | 21, -39, 66 | 1319 | 8.79 |
|  |  | 12, 9, 66 |  | 8.72 |
|  |  | 24, -12, 72 |  | 8.59 |
|  | Left | -27, -39, 66 | 340 | 8.19 |
|  |  | -15, -21, 66 |  | 7.76 |
|  |  | -15, -12, 66 |  | 5.88 |
|  |  |  |  |  |
| Occipital Cortex | Right | 21, -84, 42 | 84 | 7.08 |
|  |  | 18, -75, 21 |  | 5.89 |
|  |  | 9, -84, 45 |  | 4.94 |
|  | Left | -12, -90, 30 | 95 | 6.07 |
|  |  | -12, -87, 42 |  | 4.78 |
|  |  | -24, -96, 24 |  | 4.56 |
|  |  |  |  |  |
| Supramarginal/Inferior Parietal Cortex | Right | 63, -33, 39 | 61 | 6.57 |
|  |  | 60, -45, 45 |  | 3.56 |
|  |  |  |  |  |
| Middle Temporal Cortex | Right | 66, -33, -6 | 107 | 6.32 |
|  |  | 51, -33, -15 |  | 6.31 |
|  |  | 51, -36, -3 |  | 3.73 |
|  |  |  |  |  |
| Lingual | Right | 33, -81, -18 | 30 | 5.42 |
|  |  | 30, -78, -9 |  | 3.75 |
|  |  |  |  |  |
| Superior Temporal Cortex | Right | 51, -6, -12 | 51 | 5.06 |
|  |  | 60, -3, -9 |  | 4.46 |
|  |  |  |  |  |

Note: ITC = inferior temporal cortex; MFC = medial frontal cortex; AI = anterior insula; AMY = amygdala; vmPFC = ventromedial prefrontal cortex

Whole-brain t-scores in this table were cluster-thresholded at p < .001, to equate to p < .05, FWE. Italicized regions indicate whole-brain clusters that overlapped with ROI regions.
