# Supplementary material for: Capacity for upregulation of emotional processing in psychopathy: all you have to do is ask
Source: Soc Cogn Affect Neurosci. 2018 Sep 25;13(11):1163–76. doi: 10.1093/scan/nsy088 (PMC6234320; doi:10.1093/scan/nsy088)
Supplement: Supplementary Data [file nsy088_suppl_data.zip › scan-17-477-File023.docx]

Table s16. Regions showing differential activity between Neg_DECREASE_ and Neg_INCREASE_ trials.

| **Region** | **L/R** | **Peak coordinate** | **Cluster size** | **t-score** |
| --- | --- | --- | --- | --- |
| *Neg_INCREASE_ > Neg_DECREASE_* | | | | |
|  |  |  |  |  |
| Occipital/Cerebellum | Bilateral | -36, -36, -3 | 5063 | 6.37 |
|  |  | 6, -63, -45 |  | 5.56 |
|  |  | 18, -90, -3 |  | 5.52 |
|  |  |  |  |  |
| *Insula/Caudate/IFC/ACC/AMY* | Bilateral | -24, 30, 6 | 3046 | 5.91 |
|  |  | 21, 27, 9 |  | 5.45 |
|  |  | 27, 3, 27 |  | 5.40 |
|  |  |  |  |  |
| Occipital Cortex | Left | -18, -99, -6 | 801 | 5.57 |
|  |  | -24, -90, 6 |  | 5.23 |
|  |  | -36, -69, 30 |  | 4.11 |
|  |  |  |  |  |
| Midbrain | Bilateral | 0, -3,-3 | 82 | 4.36 |
|  |  | -3, -18, 0 |  | 3.37 |
|  |  |  |  |  |
| Dorsomedial Frontal Cortex | Bilateral | -3. 60, 33 | 52 | 4.18 |
|  |  |  |  |  |
| Superior Frontal Cortex | Left | -48, 6, 36 | 102 | 3.81 |
|  |  | -54, 0, 39 |  | 3.73 |
|  | Right | 57, 0, 39 | 36 | 3.74 |
|  |  |  |  |  |
| Postcentral Cortex | Left | -33, -21, 60 | 42 | 3.71 |
|  |  |  |  |  |
| Inferior Frontal Cortex | Left | 63, -9, 12 | 51 | 3.67 |
|  |  | 51, -6, 12 |  | 3.44 |
| *Neg_DECREASE_ > Neg_INCREASE_* |  |  |  |  |
|  |  |  |  |  |
| Angular Cortex | Right | 57, -48, 36 | 147 | 4.58 |
|  | | | | |
|  |  |  |  |  |

Note: IFC = inferior frontal cortex; ACC = anterior cingulate cortex; AMY = amygdala

Note: in Neg_INCREASE_ > Neg_DECREASE_, both anterior insula and amygdala activation occurred in left hemisphere only.

Whole-brain t-scores in this table were cluster-thresholded at p < .001, to equate to p < .05, FWE. Italicized regions indicate whole-brain clusters that overlapped with ROI regions.
