# Supplementary material for: Capacity for upregulation of emotional processing in psychopathy: all you have to do is ask
Source: Soc Cogn Affect Neurosci. 2018 Sep 25;13(11):1163–76. doi: 10.1093/scan/nsy088 (PMC6234320; doi:10.1093/scan/nsy088)
Supplement: Supplementary Data [file nsy088_suppl_data.zip › scan-17-477-File024.docx]

Table s17. Regions showing differential activity between Neg_INCREASE_ and Neg_DECREASE_ trials for Low Psychopathy and Mid Psychopathy Groups.

| **Region** | **L/R** | **Peak coordinate** | **Cluster size** | **t-score** |  |  |  |  |
| --- | --- | --- | --- | --- | --- | --- | --- | --- |
| *Mid Psychopathy > Low Psychopathy* | | | | |  |  |  |  |
|  |  |  |  |  |  |  |  |  |
| Inferior Frontal Cortex/*Anterior Insula* | Left | *-36, 24, -18* | 192 | 4.73 |  |  |  |  |
|  |  | -33, 15, -24 |  | 3.84 |  |  |  |  |
|  |  | -48, 15, -15 |  | 3.75 |  |  |  |  |
| *Anterior Insula* | *Right* | *36, 21, -18* | *41* | *4.00* |  |  |  |  |
| **Inferior Frontal Cortex** | **Right** | **42, 24, -12** | - | **3.08** |  |  |  |  |
|  |  |  |  |  |  |  |  |  |
| MFC/Superior Frontal Cortex | Right | 30, 45, -12 | 65 | 4.24 |  |  |  |  |
|  |  | 21, 54, -3 |  | 3.50 |  |  |  |  |
|  | Right | 24, -6, 45 | 62 | 3.98 |  |  |  |  |
|  |  |  |  |  |  |  |  |  |
| Cerebellum | Left | -33, -69, -33 | 107 | 4.08 |  |  |  |  |
|  |  |  |  |  |  |  |  |  |
| SFC/SMA/*ACC* | Right | *9, 33, 54* | 82 | 3.81 |  |  |  |  |
|  |  | 12, 21, 60 |  | 3.33 |  |  |  |  |
|  |  | 12, 21, 48 |  | 3.26 |  |  |  |  |
|  |  |  |  |  |  |  |  |  |
| Precentral Cortex | Left | -51, 12, 33 | 29 | 3.56 |  |  |  |  |
|  |  |  |  |  |  |  |  |  |
| Inferior Parietal Cortex | Right | 51, -39, 51 | 70 | 3.54 |  |  |  |  |
|  |  | 48, -39, 39 |  | 3.41 |  |  |  |  |
|  |  |  |  |  |  |  |  |  |
| **MFC** | **Right** | **33, 24, 48** | - | **3.60** |  |  |  |  |
|  | **Left** | **-36, 9, 54** | - | **3.14** |  |  |  |  |
|  |  |  |  |  |  |  |  |  |
| \| *Low Psychopathy > Mid Psychopathy* \| \| --- \| | | | | |  |  |  |  |
|  | | | | |  |  |  |  |
| *No significant activations* | | | | |  |  |  |  |
|  | | | | |  |  |  |  |
|  |  |  |  |  |  |  |  |  |

Note: MFC = middle frontal cortex; SFC = superior frontal cortex; SMA = supplementary motor area; ACC = anterior cingulate gyrus

Whole-brain t-scores in this table were cluster-thresholded at p < .001, to equate to p < .05, FWE. Italicized regions indicate whole-brain clusters that overlapped with ROI regions. Where overlap did not occur, small-volume correction was initiated within 10mm ROI spheres, and thresholded at *p* < .05, FWE-svc (bolded).
