# Supplementary material for: Capacity for upregulation of emotional processing in psychopathy: all you have to do is ask
Source: Soc Cogn Affect Neurosci. 2018 Sep 25;13(11):1163–76. doi: 10.1093/scan/nsy088 (PMC6234320; doi:10.1093/scan/nsy088)
Supplement: Supplementary Data [file nsy088_suppl_data.zip › scan-17-477-File035.docx]

Figure s8. Neural/subjective synchrony in each of the low, medium and high PCL-R groups (as indexed via parametric modulation of emotion ratings).


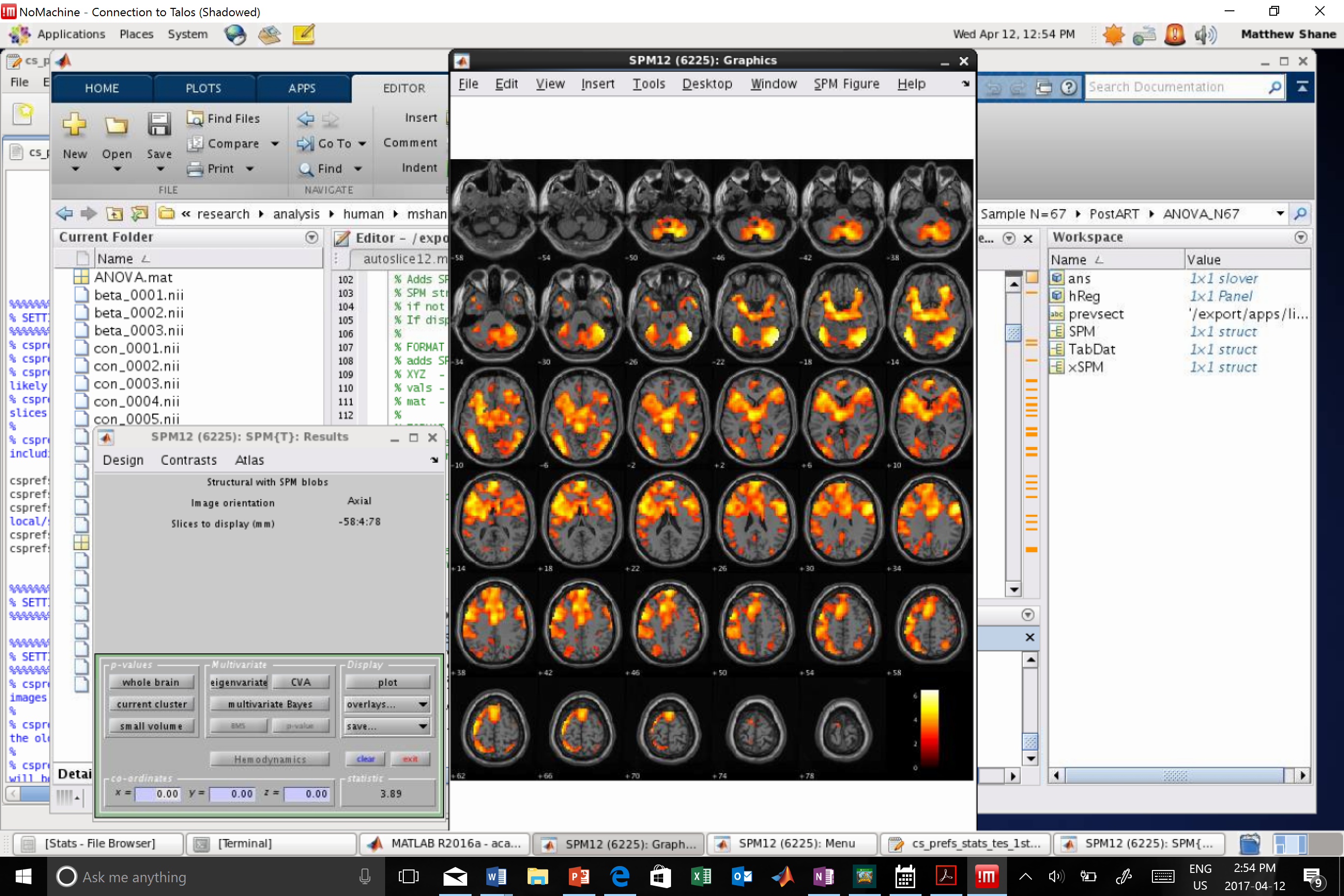

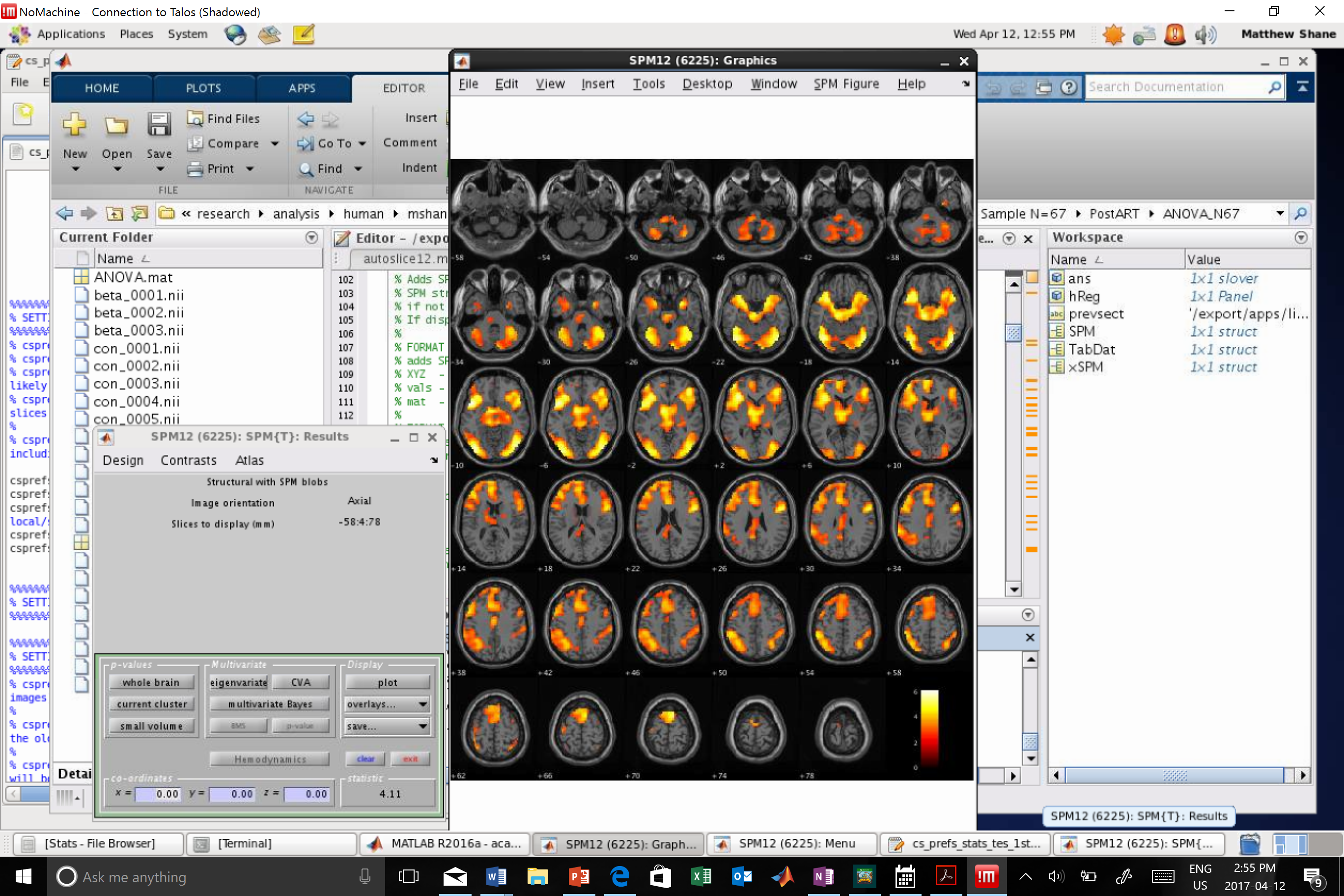

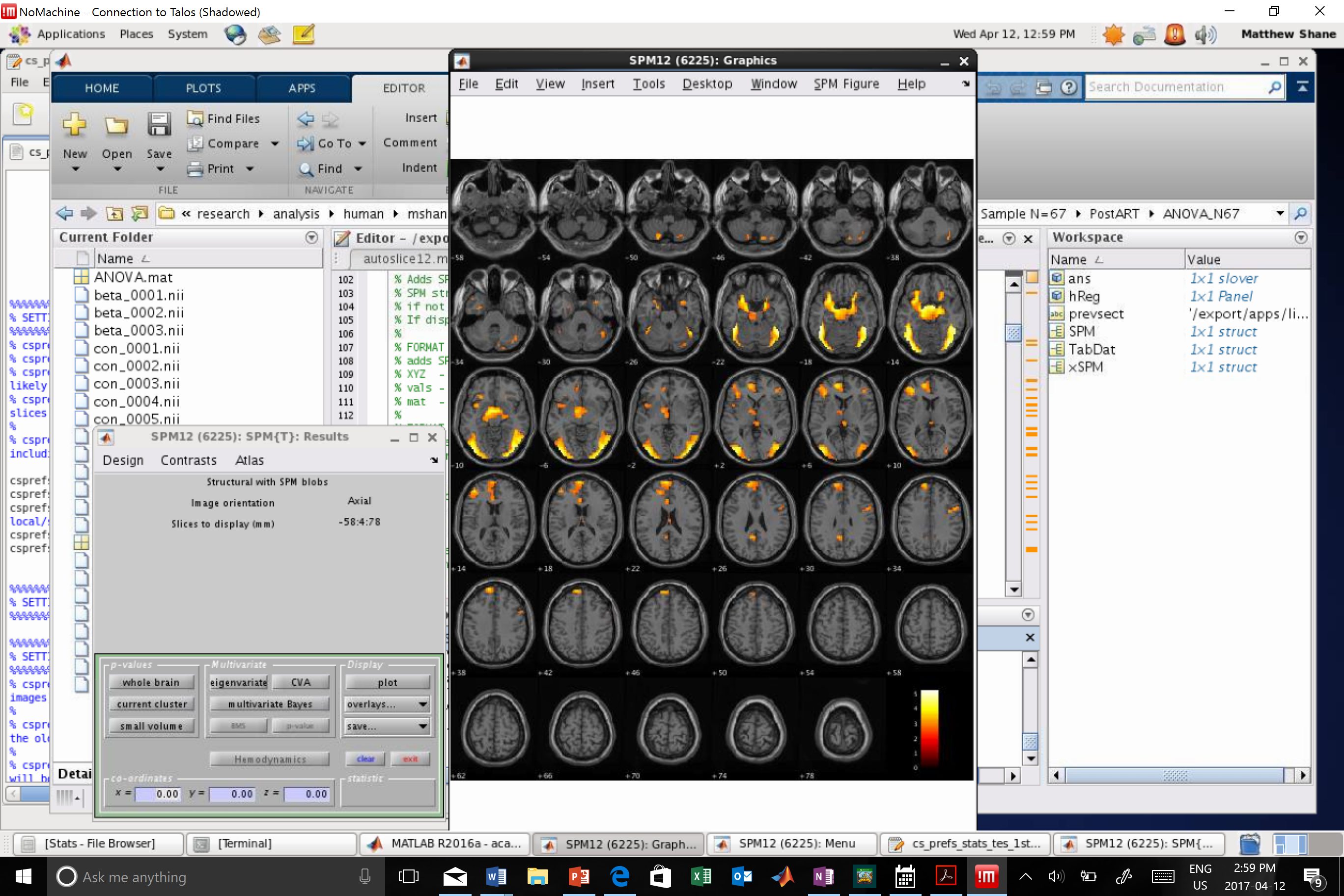


Low PCL-R

Mid PCL-R

High PCL-R
