# Supplementary material for: Capacity for upregulation of emotional processing in psychopathy: all you have to do is ask
Source: Soc Cogn Affect Neurosci. 2018 Sep 25;13(11):1163–76. doi: 10.1093/scan/nsy088 (PMC6234320; doi:10.1093/scan/nsy088)
Supplement: Supplementary Data [file nsy088_suppl_data.zip › scan-17-477-File026.docx]

Table s19. Regions showing neural responses that were parametrically modulated with subjective emotion ratings (ie. neural/subjective synchrony).

| **Region** | **L/R** | **Peak coordinate** | **Cluster size** | ***F*-score** |
| --- | --- | --- | --- | --- |
|  |  |  |  |  |
| *Midbrain/AI/IFC/AMY/mPFC/vmPFC* | Left | -12, -21, -15 | 11071 | 27.06 |
|  |  | -36, 27, 3 |  | 23.73 |
|  |  | -18, -9, -18 |  | 23.01 |
|  |  |  |  |  |
| Superior Parietal Cortex | Right | 33, -57, 57 | 248 | 11.31 |
|  |  | 63, -24, 45 |  | 9.98 |
|  |  | 39, -39, 45 |  | 7.22 |
|  |  |  |  |  |
| Posterior Cingulate Cortex | Left | -6, -51, 27 | 67 | 11.83 |
|  |  |  |  |  |
| Temporal Cortex | Right | 51, 9, -27 | 20 | 8.21 |
|  |  | 57, 3, -24 |  | 8.02 |
|  |  |  |  |  |
| Lingual/Calcarine | Bilateral | 30, -45, -9 | 64 | 15.35 |
|  |  | -27, -51, -6 | 40 | 12.45 |
|  |  | 18, -57, 15 | 106 | 11.05 |
|  |  |  |  |  |
| Occipital/Cerebrellar Cortex | Bilateral | -39, -78, -12 | 4293 | 27.83 |
|  |  | 45, -57, -24 |  | 27.74 |
|  |  | 42, -69, -18 |  | 27.43 |
|  |  |  |  |  |
|  |  |  |  |  |

Note: AI = anterior insula; IFC = inferior frontal cortex; AMY = amygdala; mPFC = medial prefrontal cortex; vmPFC = ventromedial prefrontal cortex

Whole-brain t-scores in this table were cluster-thresholded at p < .001, to equate to p < .05, FWE. Italicized regions indicate whole-brain clusters that overlapped with ROI regions.
