# Supplementary material for: Capacity for upregulation of emotional processing in psychopathy: all you have to do is ask
Source: Soc Cogn Affect Neurosci. 2018 Sep 25;13(11):1163–76. doi: 10.1093/scan/nsy088 (PMC6234320; doi:10.1093/scan/nsy088)
Supplement: Supplementary Data [file nsy088_suppl_data.zip › scan-17-477-File015.docx]

Table s8. Regions showing differential activity between Neg_WATCH_ and Neut_WATCH_ trials in Low Psychopathy Group.

| **Region** | **L/R** | **Peak coordinate** | **Cluster size** | **t-score** |
| --- | --- | --- | --- | --- |

| *Neg_WATCH_ > Neut_WATCH_* | | | | |
| --- | --- | --- | --- | --- |
|  |  |  |  |  |
| Occipital Cortex | Right | 48, -63, -9 | 687 | 8.33 |
|  |  | 45, -54, -15 |  | 7.54 |
|  |  | 33, -93, -6 |  | 5.14 |
|  | Left | -45, -75, -9 | 821 | 7.74 |
|  |  | -45, -51, -18 |  | 7.70 |
|  |  | -27, -99, 3 |  | 3.99 |
|  |  |  |  |  |
| *Amygdala/NAcc/Thalamus* | Bilateral | 9, -6, -15 | 785 | 5.49 |
|  |  | *21, -3, -18* |  | *4.98* |
|  |  | -6, -6, -12 |  | 4.75 |
|  |  |  |  |  |
| *Insula/OFC* | Right | 51, 42, 0 | 179 | 5.02 |
|  |  | *42, 33, -15* |  | *4.28* |
|  |  | 24, 27, -15 |  | 4.11 |
|  |  |  |  |  |
| Dorsomedial Prefrontal Cortex | Left | -6, 51, 18 | 223 | 4.74 |
|  |  | -9, 63, 24 |  | 4.26 |
|  |  | -6, 51, 42 |  | 3.42 |
|  |  |  |  |  |
| Inferior Frontal Cortex | Left | -42, 36, 6 | 185 | 4.62 |
|  |  |  |  |  |
| Precentral Cortex | Right | 45, 6, 24 | 102 | 4.67 |
|  | Left | -45, 3, 30 | 42 | 3.81 |
|  |  |  |  |  |
| Superior Parietal Cortex | Right | 33, -42, 45 | 119 | 4.60 |
|  |  | 33, -54, 60 |  | 3.76 |
|  | Left | -30, -63, 60 | 51 | 4.07 |
|  |  |  |  |  |
| **Ventromedial Prefrontal Cortex** | **Left** | **-6, 45, 6** | **-** | **3.03** |
|  |  |  |  |  |

| *Neut_WATCH_ > Neg_WATCH_* |  |  |  |  |
| --- | --- | --- | --- | --- |
|  |  |  |  |  |
| Lingual/Vermis/Calcarine | Right | 30, -45, -9 | 656 | 7.17 |
|  |  | 24, -39, -15 |  | 7.06 |
|  |  | 15, -51, 12 |  | 6.95 |
|  | Left | -15, -48, 6 | 478 | 7.03 |
|  |  | -24, -45, -9 |  | 6.80 |
|  |  |  |  |  |
| Precuneus | Bilateral | 9, -54, 4 | 51 | 3.73 |
|  |  | -3, -48, 48 |  | 3.63 |
|  |  |  |  |  |
| Superior Temporal Cortex | Right | 57, -15, 3 | 33 | 3.46 |
|  |  | 66, -18, 0 |  | 3.39 |
|  |  | 63, -12, -6 |  | 3.22 |
|  |  |  |  |  |

Note: NAcc = nucleus accumbens; OFC = orbitofrontal cortex.

Whole-brain t-scores in this table were cluster-thresholded at p < .001, to equate to p < .05, FWE. Italicized regions indicate whole-brain clusters that overlapped with ROI regions. Where overlap did not occur, small-volume correction was initiated within 10mm ROI spheres, and thresholded at *p* < .05, FWE-svc (bolded).
