# Supplementary material for: Capacity for upregulation of emotional processing in psychopathy: all you have to do is ask
Source: Soc Cogn Affect Neurosci. 2018 Sep 25;13(11):1163–76. doi: 10.1093/scan/nsy088 (PMC6234320; doi:10.1093/scan/nsy088)
Supplement: Supplementary Data [file nsy088_suppl_data.zip › scan-17-477-File016.docx]

Table s9. Regions showing differential activity between Neg_WATCH_ and Neut_WATCH_ trials in Mid Psychopathy Group.

| **Region** | | **L/R** | **Peak coordinate** | **Cluster size** | **t-score** |
| --- | --- | --- | --- | --- | --- |
| *Neg_WATCH_ > Neut_WATCH_* | | | | | |
|  |  | |  |  |  |
| Occipital Cortex/Cerebellar | Left | | -48, -72, -9 | 601 | 6.47 |
|  |  | | -42, -60, -15 |  | 5.98 |
|  |  | | -36, -72, -30 |  | 3.96 |
|  | Right | | 48, -66, -9 | 250 | 5.55 |
|  |  | | 48, -45, -15 |  | 4.49 |
|  |  | |  |  |  |
| Supramarginal/Angular Gyrus | Left | | -42, -39, 42 | 409 | 5.48 |
|  |  | |  |  |  |
| *Hippocampal/Thalamus/Amygdala* | Bilateral | | -6, 0, -6 | 396 | 4.56 |
|  |  | | -12, -12, -12 |  | 4.50 |
|  |  | | 15, 0, 6 |  | 4.41 |
|  |  | |  |  |  |
| Middle/Lateral Frontal Cortex | Left | | -48, 45, -3 | 261 | 4.20 |
|  |  | | -42, 36, 15 |  | 4.05 |
|  |  | | -27, 60, 9 |  | 3.94 |
|  |  | |  |  |  |
| Inferior Frontal Cortex | Right | | 51, 9, 36 | 81 | 4.14 |
|  |  | |  |  |  |
| *Insula/OFC* | Left | | *-33, 24, -3* | *57* | *3.91* |
|  |  | | *-33, 21, -15* |  | *3.38* |
|  |  | |  |  |  |
| Supramarginal/Angular Gyrus | Right | | 36, -51, 48 | 60 | 3.90 |
|  |  | | 42, -39, 45 |  | 3.79 |
|  |  | |  |  |  |
| **Insula** | **Right** | | **48, 21, -12** | **-** | **3.37** |
|  |  | |  |  |  |
| *Neut_WATCH_ > Neg_WATCH_* |  | |  |  |  |
|  |  | |  |  |  |
| Lingual/Vermis/Calcarine | Bilateral | | 18, -57, 15 | 1769 | 8.83 |
|  |  | | 30, -45, -9 |  | 7.99 |
|  |  | | -27, -45, -9 |  | 7.19 |
|  |  | |  |  |  |
| Superior Temporal Cortex | Right | | 57, -12, -30 | 211 | 4.87 |
|  |  | | 45, -30, 15 |  | 4.11 |
|  |  | | 54, -27, 6 |  | 4.09 |
|  |  | |  |  |  |
| Occipital Cortex | Right | | 42, -78, 30 | 83 | 4.78 |
|  |  | |  |  |  |

Note: OFC = orbitofrontal cortex

Whole-brain t-scores in this table were cluster-thresholded at p < .001, to equate to p < .05, FWE. Italicized regions indicate whole-brain clusters that overlapped with ROI regions. Where overlap did not occur, small-volume correction was initiated within 10mm ROI spheres, and thresholded at *p* < .05, FWE-svc (bolded).
