# Supplementary material for: Capacity for upregulation of emotional processing in psychopathy: all you have to do is ask
Source: Soc Cogn Affect Neurosci. 2018 Sep 25;13(11):1163–76. doi: 10.1093/scan/nsy088 (PMC6234320; doi:10.1093/scan/nsy088)
Supplement: Supplementary Data [file nsy088_suppl_data.zip › scan-17-477-File009.docx]

Table s2. Regions showing differential activity between Neg_INCREASE_ and Neg_WATCH_ trials.

| **Region** | **L/R** | **Peak coordinate** | **Cluster size** | **t-score** |
| --- | --- | --- | --- | --- |
| *Neg_INCREASE_ > Neg_WATCH_* | | | | |
|  |  |  |  |  |
| *SMA/ACC/AI/AMY/IFC/vmPFC/mPFC/OFC* | Bilateral | 0, 9, 63 | 14225 | 7.50 |
|  |  | 36, -57, -30 |  | 6.90 |
|  |  | *-48, 12, -3* |  | *6.73* |
|  |  |  |  |  |
| Middle Frontal Cortex | Right | 57, 3, 42 | 70 | 54.59 |
|  |  |  |  |  |
| *Anterior Insula* | Right | *48, 18, -12* | 625 | 4.53 |
|  |  | *36, 18, -12* |  | 4.51 |
|  |  | 45, 9, -3 |  | 4.34 |
|  | | | | |
| *Neg_WATCH_ > Neg_INCREASE_* |  |  |  |  |
|  |  |  |  |  |
| *No significant clusters* | | | | |
|  |  |  |  |  |

Note: SMA = supplementary motor area; ACC = anterior cingulate cortex; AI = anterior insula; AMY = amygdala; IFC = inferior frontal cortex; vmPFC = ventromedial prefrontal cortex; mPFC = medial prefrontal cortex; OFC = orbitofrontal cortex

Whole-brain t-scores in this table were cluster-thresholded at p < .001, to equate to p < .05, FWE. Italicized regions indicate whole-brain clusters that overlapped with ROI regions.
