# Supplementary material for: Capacity for upregulation of emotional processing in psychopathy: all you have to do is ask
Source: Soc Cogn Affect Neurosci. 2018 Sep 25;13(11):1163–76. doi: 10.1093/scan/nsy088 (PMC6234320; doi:10.1093/scan/nsy088)
Supplement: Supplementary Data [file nsy088_suppl_data.zip › scan-17-477-File025.docx]

Table s18. Regions showing differential activity between Neg_INCREASE_ and Neg_DECREASE_ trials for Low Psychopathy and High Psychopathy Groups.

| **Region** | **L/R** | **Peak coordinate** | **Cluster size** | **t-score** |
| --- | --- | --- | --- | --- |
| *High Psychopathy > Low Psychopathy* | | | | |
|  |  |  |  |  |
| Medial Prefrontal Cortex | Left | -24, 30, 3 | 85 | 3.82 |
|  |  | -21, 21, 6 |  | 3.58 |
|  |  | -27, 12, 21 |  | 3.49 |
|  |  |  |  |  |
| MFC | Left | -42, -9, 33 | 45 | 3.58 |
|  |  | -39, 0, 33 |  | 3.41 |
| **MFC** | **Left** | **-42, 3, 36** | **-** | **3.10** |
|  |  |  |  |  |
| **ACC** | **Left** | **-18, 18, 18** | **-** | **2.79** |
|  | **Right** | **15, 18, 48** | **-** | **3.30** |
|  |  |  |  |  |
| **Amygdala** | **Left** | **-24, -6, -21** | **-** | **2.80** |
|  |  |  |  |  |
| **Anterior Insula** | **Left** | **-33, 24, -18** | **-** | **2.71^†^** |
|  |  |  |  |  |
| \| *Low Psychopathy > High Psychopathy* \| \| --- \| | | | | |
|  |  |  |  |  |
| *No significant activations* | | | | |
|  |  |  |  |  |

Note: ACC = anterior cingulate cortex; MFC = middle frontal cortex

Whole-brain t-scores in this table were cluster-thresholded at p < .001, to equate to p < .05, FWE. Italicized regions indicate whole-brain clusters that overlapped with ROI regions. Where overlap did not occur, small-volume correction was initiated within 10mm ROI spheres, and thresholded at *p* < .05, FWE-svc (bolded). **^†^** *p* = .059, FWE-svc.
