# Supplementary material for: Capacity for upregulation of emotional processing in psychopathy: all you have to do is ask
Source: Soc Cogn Affect Neurosci. 2018 Sep 25;13(11):1163–76. doi: 10.1093/scan/nsy088 (PMC6234320; doi:10.1093/scan/nsy088)
Supplement: Supplementary Data [file nsy088_suppl_data.zip › scan-17-477-File019.docx]

Table s12. Regions showing differential activity between Neg_INCREASE_ and Neg_WATCH_ trials in Low Psychopathy Group.

| **Region** | **L/R** | **Peak coordinate** | **Cluster size** | **t-score** |
| --- | --- | --- | --- | --- |
| *Neg_INCREASE_ > Neg_WATCH_* | | | | |
|  |  |  |  |  |
| Angular Gyrus | Left | -51, -48, 48 | 298 | 4.59 |
|  |  | -57, -48, 36 |  | 4.42 |
|  |  |  |  |  |
| Frontal Operculum | Left | -48, 12, -3 | 179 | 4.58 |
|  |  | -51, 9, 6 |  | 3.97 |
|  |  | -33, 24, 3 |  | 3.74 |
|  |  |  |  |  |
| Cerebellum | Right | 33, -48, -48 | 258 | 4.42 |
|  |  | 42, -60, -42 |  | 4.14 |
|  |  | 33, -54, -30 |  | 4.05 |
|  |  |  |  |  |
| Superior Frontal Cortex/SMA | Bilateral | -21, 18, 60 | 212 | 4.41 |
|  |  | -6, 3, 60 |  | 4.33 |
|  |  |  |  |  |
| Middle Frontal Cortex | Left | -30, 57, 18 | 93 | 4.20 |
|  |  |  |  |  |
| Lateral Frontal Cortex | Left | -42, 30, 39 | 146 | 3.96 |
|  |  | -45, 3, 51 |  | 3.86 |
|  |  |  |  |  |
| **Insula** | **Left** | **-42, 18, -6** | - | **3.74** |
|  | **Right** | **45, 18, -9** | - | **2.89** |
|  |  |  |  |  |
| *Neg_WATCH_ > Neg_INCREASE_* |  |  |  |  |
|  |  |  |  |  |
| Middle Temporal Cortex | Right | 48, 3, -27 | 64 | 4.50 |
|  |  | 60, -18, -24 |  | 3.40 |
|  |  | 54, -12, -27 |  | 3.39 |
|  |  |  |  |  |
| **Amygdala** | **Right** | **27, 3, -27** | **-** | **2.82** |
|  |  |  |  |  |

Note: SMA = supplementary motor area

Whole-brain t-scores in this table were cluster-thresholded at p < .001, to equate to p < .05, FWE. Italicized regions indicate whole-brain clusters that overlapped with ROI regions. Where overlap did not occur, small-volume correction was initiated within 10mm ROI spheres, and thresholded at *p* < .05, FWE-svc (bolded).
