# Supplementary material for: Capacity for upregulation of emotional processing in psychopathy: all you have to do is ask
Source: Soc Cogn Affect Neurosci. 2018 Sep 25;13(11):1163–76. doi: 10.1093/scan/nsy088 (PMC6234320; doi:10.1093/scan/nsy088)
Supplement: Supplementary Data [file nsy088_suppl_data.zip › scan-17-477-File027.docx]

Table s20. Regions showing correlations between PCL-R scores and neural/subjective synchrony (indexed via parametric modulations with subjective emotion ratings).

| **Region** | **L/R** | **Peak coordinate** | **Cluster size** | ***t*-score** |
| --- | --- | --- | --- | --- |
|  | | | | |
| *Positive Correlations* |  |  |  |  |
| Inferior Frontal Cortex | Left | -51, 18, 18 | 39 | 4.15 |
|  |  |  |  |  |
| Precentral Cortex/SMA | Left | -42, -12, 60 | 56 | 3.71 |
|  |  | -36, -15, 66 |  | 3.49 |
|  |  |  |  |  |
|  |  |  |  |  |

Note: SMA = supplementary motor area

Whole-brain t-scores in this table were cluster-thresholded at p < .001, to equate to p < .05, FEW.
