# Supplementary material for: Capacity for upregulation of emotional processing in psychopathy: all you have to do is ask
Source: Soc Cogn Affect Neurosci. 2018 Sep 25;13(11):1163–76. doi: 10.1093/scan/nsy088 (PMC6234320; doi:10.1093/scan/nsy088)
Supplement: Supplementary Data [file nsy088_suppl_data.zip › scan-17-477-File034.docx]

Figure s7. Neg_INCREASE_ > Neg_DECREASE_ pooled effects


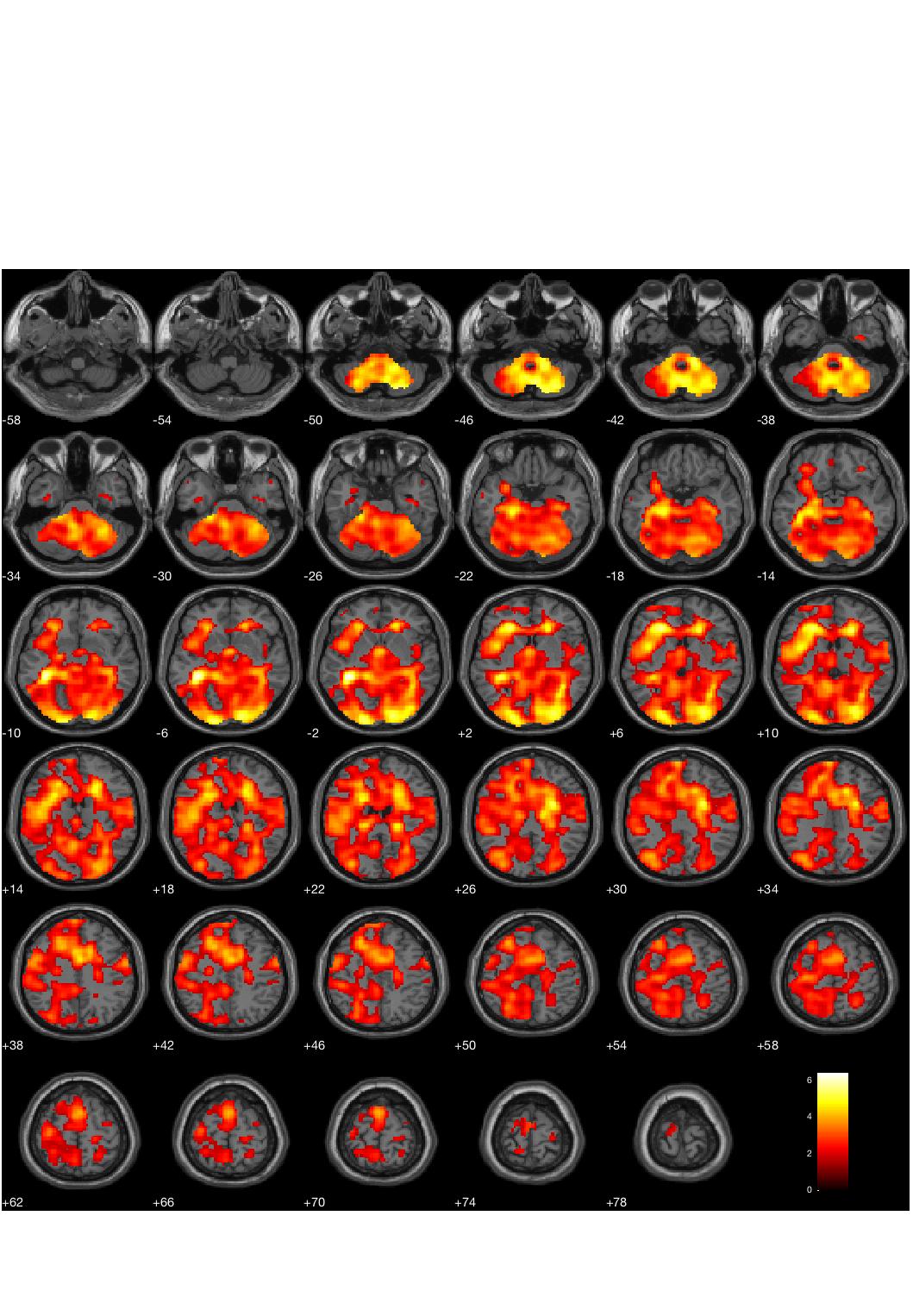


Note: image is shown at the .05 significance level.
