# Supplementary material for: Capacity for upregulation of emotional processing in psychopathy: all you have to do is ask
Source: Soc Cogn Affect Neurosci. 2018 Sep 25;13(11):1163–76. doi: 10.1093/scan/nsy088 (PMC6234320; doi:10.1093/scan/nsy088)
Supplement: Supplementary Data [file nsy088_suppl_data.zip › scan-17-477-File021.docx]

Table s14. Regions showing differential activity between Neg_INCREASE_ and Neg_WATCH_ trials in High Psychopathy Group.

| **Region** | **L/R** | **Peak coordinate** | **Cluster size** | **t-score** |
| --- | --- | --- | --- | --- |
| *Neg_INCREASE_ > Neg_WATCH_* | | | | |
|  |  |  |  |  |
| SMA/Anterior Cingulate | Bilateral | 0, 12, 63 | 619 | 5.10 |
|  |  | -6, 27, 42 |  | 4.30 |
|  |  | 15, 24, 39 |  | 3.82 |
|  |  |  |  |  |
| Precentral/Midfrontal Cortex | Left | -45, 3, 42 | 211 | 4.76 |
|  |  | -33, 21, 36 |  | 3.68 |
|  |  |  |  |  |
| Cerebellum/Occipital Pole | Bilateral | 30, -57, -36 | 1226 | 4.44 |
|  |  | -15, -93, -3 |  | 4.42 |
|  |  | 15, -90, -9 |  | 4.07 |
|  |  |  |  |  |
| Thalamus/Hippocampus | Bilateral | -6, -21, 12 | 176 | 4.27 |
|  |  | -6, -24, -3 |  | 3.77 |
|  |  |  |  |  |
| Angular Cortex/TPJ | Left | -36, -60, 24 | 149 | 4.17 |
|  |  | -54, -48, 21 |  | 3.61 |
|  |  | -57, -39, 24 |  | 3.57 |
|  |  |  |  |  |
| *Anterior Insula* | Left | *-30, 18, -12* | 305 | 4.13 |
|  |  | -27, 27, 3 |  | 4.00 |
|  |  | -30, 39, -12 |  | 3.78 |
|  |  |  |  |  |
| Cerebellum | Left | -39, -51, -36 | 107 | 3.88 |
|  |  | -21, -63, -30 |  | 3.48 |
|  |  | -21, -36, -33 | 38 | 3.66 |
|  |  |  |  |  |
| Superior Temporal Cortex | Right | 48, -27, -6 | 32 | 3.73 |
|  |  |  |  |  |
|  |  | -48, -45, 48 | 28 | 3.42 |
|  |  | -45, -39, 39 |  | 3.38 |
|  |  |  |  |  |
| *Neg_WATCH_ > Neg_INCREASE_* |  |  |  |  |
|  |  |  |  |  |
| *No significant clusters* | | | | |
|  |  |  |  |  |

Note: SMA = supplementary motor area; TPJ = temporoparietal junction

Whole-brain t-scores in this table were cluster-thresholded at p < .001, to equate to p < .05, FWE. Italicized regions indicate whole-brain clusters that overlapped with ROI regions.
