# Supplementary material for: Capacity for upregulation of emotional processing in psychopathy: all you have to do is ask
Source: Soc Cogn Affect Neurosci. 2018 Sep 25;13(11):1163–76. doi: 10.1093/scan/nsy088 (PMC6234320; doi:10.1093/scan/nsy088)
Supplement: Supplementary Data [file nsy088_suppl_data.zip › scan-17-477-File014.docx]

Table s7. Regions showing differential activity between Neg_WATCH_ and Neut_WATCH_ trials.

| **Region** | **L/R** | **Peak coordinate** | **Cluster size** | **t-score** |
| --- | --- | --- | --- | --- |
| *Neg_WATCH_ > Neut_WATCH_* | | | | |
|  |  |  |  |  |
| Occipital Cortex | Right | 48, -63, -9 | 497 | 9.60 |
|  |  | 33, -93, -6 |  | 3.64 |
|  | Left | -45, -72, -9 | 636 | 8.89 |
|  |  | -42, -57, -15 |  | 7.62 |
|  |  |  |  |  |
| Dorsomedial Prefrontal Cortex | Left | -9, 60, 27 | 316 | 5.97 |
|  |  | -21, -72, -51 |  | 6.03 |
|  |  |  |  |  |
| *Amygdala/NAcc/Thalamus* | Bilateral | -6, -3, -9 | 846 | 5.92 |
|  |  | 6, -6, -12 |  | 5.68 |
|  |  | *-15, -9, -12* |  | *5.25* |
|  |  |  |  |  |
| Cerebellum | Left | -18, -72, -51 | 40 | 4.73 |
|  |  |  |  |  |
| *Orbitofrontal/Insula* | Right | 51, 42, 3 | 164 | 4.70 |
|  |  | 51, 39, -9 |  | 4.43 |
|  |  | *36, 27, -18* |  | *3.79* |
|  |  |  |  |  |
| Inferior Frontal Cortex | Right | 51, 9, 27 | 126 | 4.64 |
|  |  |  |  |  |
| Lateral Frontal | Left | -42, 36, 12 | 62 | 4.13 |
|  |  |  |  |  |
| Inferior Parietal Cortex | Right | 39, -39, 45 | 75 | 4.01 |
|  |  | 33, -54, 57 |  | 3.48 |
|  | Left | -42, -36, 42 | 50 | 3.92 |
|  |  |  |  |  |
| **Insula** | **Left** | **-30, 21, -15** | - | **3.61** |
|  |  |  |  |  |
| *Neut_WATCH_ > Neg_WATCH_* |  |  |  |  |
|  |  |  |  |  |
| Lingual/Vermis/Calcarine | Bilateral | 15, -54, 15 | 3645 | 10.38 |
|  |  | 30, -45, -9 |  | 10.38 |
|  |  | -27, -45, -9 |  | 10.14 |
|  |  |  |  |  |
| Postcentral Gyrus | Right | 6, -51, 48 | 5.42 | 5.53 |
|  |  | 6, -36, 60 |  | 4.34 |
|  |  | 27, -18, 72 |  | 3.66 |
|  |  |  |  |  |
| Precuneus | Left | -39, -78, 27 | 105 | 5.19 |
|  |  |  |  |  |
| Superior Temporal Cortex | Left | -51, -33, 12 | 338 | 4.48 |
|  |  | -60, -24, 3 |  | 4.05 |
|  |  | -54, -9, -6 |  | 3.85 |
|  |  |  |  |  |
| Postcentral Cortex | Left | -24, -27, 63 | 48 | 3.83 |
|  |  |  |  |  |

Note: NAcc = nucleus accumbens

Whole-brain t-scores in this table were cluster-thresholded at p < .001, to equate to p < .05, FWE. Italicized regions indicate whole-brain clusters that overlapped with ROI regions. Where overlap did not occur, small-volume correction was initiated within 10mm ROI spheres, and thresholded at *p* < .05, FWE-svc (bolded).
