# Supplementary material for: Capacity for upregulation of emotional processing in psychopathy: all you have to do is ask
Source: Soc Cogn Affect Neurosci. 2018 Sep 25;13(11):1163–76. doi: 10.1093/scan/nsy088 (PMC6234320; doi:10.1093/scan/nsy088)
Supplement: Supplementary Data [file nsy088_suppl_data.zip › scan-17-477-File020.docx]

Table s13. Regions showing differential activity between Neg_INCREASE_ and Neg_WATCH_ trials in Mid Psychopathy Group.

| **Region** | | **L/R** | **Peak coordinate** | **Cluster size** | **t-score** |
| --- | --- | --- | --- | --- | --- |
| *Neg_INCREASE_ > Neg_WATCH_* | | | | | |
|  |  | |  |  |  |
| *AI/AMY/mPFC/vmPFC/SMA* | Left | | -3, 3, 72 | 3798 | 5.72 |
|  |  | | -39, 15, -6 |  | 5.37 |
|  |  | | -48, 12, -6 |  | 5.34 |
|  |  | |  |  |  |
| *AI/OFC* | Right | | *48, 18, -12* | *333* | *5.76* |
|  |  | | *36, 15, -12* |  | 3.85 |
|  |  | | 39, 33, 3 | - | 3.69 |
|  |  | |  |  |  |
| Inferior Parietal/Precuneus | Left | | -54, -54, 42 | 532 | 5.50 |
|  |  | | -57, -45, 45 |  | 5.46 |
|  |  | | -42, -63, 54 |  | 4.88 |
|  |  | |  |  |  |
| Cerebellum | Right | | 39, -72, -42 | 497 | 4.79 |
|  |  | | 36, -60, -30 |  | 4.65 |
|  |  | | 30, -81, -24 |  | 3.99 |
|  | Left | | -39, -60, -30 | 349 | 4.58 |
|  |  | | -39, -66, -45 |  | 4.45 |
|  |  | | -27, -75, -24 |  | 3.62 |
|  |  | |  |  |  |
| Parahippocampal Cortex | Left | | -6, -18, -18 | 200 | 4.25 |
|  |  | | -12, -24, -27 |  | 4.15 |
|  |  | | -24, -21, -18 |  | 3.84 |
|  | Right | | 18, -24, -18 | 68 | 3.96 |
|  |  | | 33, -18, -18 |  | 3.54 |
|  |  | |  |  |  |
|  |  | |  |  |  |
| Thalamus | Bilateral | | -3, -3, 3 | 117 | 4.22 |
|  |  | | 3, 0, -3 |  | 4.08 |
|  |  | | -15, -6, 6 |  | 3.25 |
|  |  | |  |  |  |
| Precuneus/Occipital Cortex | Bilateral | | -6, -75, 30 | 302 | 4.02 |
|  |  | | -3, -81, 48 |  | 4.00 |
|  |  | | 18, -75, 18 |  | 3.63 |
|  |  | |  |  |  |
| Midcingulate/SMA | Right | | 15, -24, 51 | 30 | 4.00 |
|  |  | |  |  |  |
| Precentral Cortex | Right | | 60, 0, 39 | 53 | 3.87 |
|  |  | | 51, -3, 51 |  | 3.65 |
| Middle Temporal Cortex | Left | | -63, -27, -6 | 42 | 3.79 |
|  |  | |  |  |  |
| Angular Cortex | Right | | -42, -36, 27 | 31 | 3.75 |
|  |  | |  |  |  |
| Postcentral Cortex | Right | | 48, -36, 33 | 65 | 3.72 |
|  |  | | 63, -30, 45 |  | 3.71 |
|  |  | |  |  |  |
| Caudate | Right | | 21, 6, 18 | 43 | 3.43 |
|  |  | | 24, 15, 12 |  | 3.41 |
|  |  | |  |  |  |
| *Neg_WATCH_ > Neg_INCREASE_* |  | |  |  |  |
| *No significant activations.* | | | | | |
|  |  | |  |  |  |

Note: AI = anterior insula; AMY = amygdala; mPFC = medial prefrontal cortex; vmPFC = ventromedial prefrontal cortex; SMA = supplementary motor area; OFC = orbitofrontal cortex; SMA = supplementary motor area

Whole-brain t-scores in this table were cluster-thresholded at p < .001, to equate to p < .05, FWE. Italicized regions indicate whole-brain clusters that overlapped with ROI regions.
