# Supplementary material for: Capacity for upregulation of emotional processing in psychopathy: all you have to do is ask
Source: Soc Cogn Affect Neurosci. 2018 Sep 25;13(11):1163–76. doi: 10.1093/scan/nsy088 (PMC6234320; doi:10.1093/scan/nsy088)
Supplement: Supplementary Data [file nsy088_suppl_data.zip › scan-17-477-File010.docx]

Table s3. Regions showing differential activity between Neg_DECREASE_ and Neg_WATCH_ trials.

| **Region** | **L/R** | **Peak coordinate** | **Cluster size** | **t-score** |
| --- | --- | --- | --- | --- |
| *Neg_DECREASE_ > Neg_WATCH_* | | | | |
|  |  |  |  |  |
| Inferior Parietal | Left | -57, -51, 42 | 710 | 8.42 |
|  |  | -42, -63, 54 |  | 5.70 |
|  |  |  |  |  |
| Supramarginal Cortex | Right | 57, -45, 36 | 572 | 6.56 |
|  |  |  |  |  |
| Mid Temporal Cortex | Left | -63, -30, -9 | 225 | 5.59 |
|  |  | -66, -39, -6 |  | 5.58 |
|  | Right | 63, -30, -9 | 186 | 5.36 |
|  |  |  |  |  |
| *Anterior Insula/OFC* | Left | -51, 15, -6 | 297 | 5.10 |
|  |  | *-33, 18, -9* |  | 4.02 |
|  |  |  |  |  |
| Middle/Superior Frontal Cortex/SMA | Left | -42, 21, 45 | 1739 | 5.01 |
|  |  | -3, 27, 60 |  | 4.95 |
|  |  | 9, 27, 39 |  | 4.83 |
|  |  |  |  |  |
| *Inferior/Middle OFC/Insula* | Right | *48, 21, -9* | 517 | 4.91 |
|  |  | 54, 18, 3 |  | 4.54 |
|  |  | 33, 54, -3 |  | 4.04 |
|  |  |  |  |  |
| Lateral Frontal Cortex | Right | 36, 48, 18 | 354 | 4.64 |
|  |  | 42, -36, 30 |  | 4.46 |
|  |  | 45, 18, 42 |  | 4.38 |
|  |  |  |  |  |
| Cerebellum | Left | -42, -51, -36 | 52 | 4.47 |
|  | Right | 36, -57, -30 | 83 | 3.88 |
|  |  | 24, -75, -27 |  | 3.57 |
|  |  |  |  |  |
| Precuneus/Cuneus | Right | 9, -63, 48 | 97 | 4.33 |
|  |  | 6, -84, 42 |  | 3.36 |
|  |  | -6, -57, 42 |  | 3.24 |
|  |  |  |  |  |
| Mid Cingulate Cortex | Right | 3, -21, 39 | 77 | 3.94 |
|  |  | 0, -24, 27 |  | 3.79 |
|  |  |  |  |  |
| *Neg_WATCH_ > Neg_DECREASE_* |  |  |  |  |
|  |  |  |  |  |
| *No significant clusters* | | | | |
|  |  |  |  |  |

Note: SMA = supplementary motor area; middle OFC = middle orbitofrontal cortex

Whole-brain t-scores in this table were cluster-thresholded at p < .001, to equate to p < .05, FWE. Italicized regions indicate whole-brain clusters that overlapped with ROI regions. Where overlap did not occur, small-volume correction was initiated within 10mm ROI spheres, and thresholded at *p* < .05, FWE-svc (bolded).
