# Supplementary material for: Capacity for upregulation of emotional processing in psychopathy: all you have to do is ask
Source: Soc Cogn Affect Neurosci. 2018 Sep 25;13(11):1163–76. doi: 10.1093/scan/nsy088 (PMC6234320; doi:10.1093/scan/nsy088)
Supplement: Supplementary Data [file nsy088_suppl_data.zip › scan-17-477-File022.docx]

Table s15. Regions showing differential activity between Neg_INCREASE_ and Neg_WATCH_ trials for High Psychopathy compared to Low/Mid Psychopathy Groups.

| **Region** | **L/R** | **Peak coordinate** | **Cluster size** | **t-score** |
| --- | --- | --- | --- | --- |
| *Neg_INCREASE_ > Neg_WATCH_  for Low/Mid Psychopathy Groups > High Psychopathy Group* | | | | |
|  |  |  |  |  |
| Occipital Cortex | Left | -15, -93, -6 | 32 | 3.79 |
|  | | | | |
| *Neg_INCREASE_ > Neg_WATCH_  for High Psychopathy Group > Low/Mid Psychopathy Groups* | | | | |
|  |  |  |  |  |
| No significant activations | | | | |
|  |  |  |  |  |
|  | | | | |

Whole-brain t-scores in this table were cluster-thresholded at p < .001, to equate to p < .05, FWE. Italicized regions indicate whole-brain clusters that overlapped with ROI regions. Where overlap did not occur, small-volume correction was initiated within 10mm ROI spheres, and thresholded at *p* < .05, FWE-svc (bolded).
