# Supplementary material for: Capacity for upregulation of emotional processing in psychopathy: all you have to do is ask
Source: Soc Cogn Affect Neurosci. 2018 Sep 25;13(11):1163–76. doi: 10.1093/scan/nsy088 (PMC6234320; doi:10.1093/scan/nsy088)
Supplement: Supplementary Data [file nsy088_suppl_data.zip › scan-17-477-File012.docx]

Table s5. Regions showing differential activity between Low Psychopathy and High Psychopathy Groups.

| **Region** | **L/R** | **Peak coordinate** | **Cluster size** | **t-score** |
| --- | --- | --- | --- | --- |
| *Low Psychopathy Group > High Psychopathy Group* | | | | |
|  |  |  |  |  |
| *Parahipp/Sup Parietal/AI/AMY/vmPFC* | Bilateral | 33, -27, -21 | 32 626 | 17.38 |
|  |  | -21, -69, 33 |  | 15.82 |
|  |  | 27, -63, 30 |  | 15.79 |
|  |  |  |  |  |
| *High Psychopathy Group > Low Psychopathy Group* | | | | |
|  |  |  |  |  |
| Occipital Cortex | Right | 27, -96, 9 | 296 | 10.78 |
|  |  | 33, -90, 0 |  | 8.85 |
|  |  | 9, -84, -15 |  | 3.94 |
|  |  |  |  |  |
| SMA | Bilateral | -6, 24, 66 | 759 | 9.84 |
|  |  | -9, 15, 69 |  | 9.68 |
|  |  | 12, 9, 69 |  | 9.41 |
|  |  |  |  |  |
| Superior Parietal Cortex | Right | 48, -48, 60 | 85 | 8.31 |
|  |  | 57, -39, 54 |  | 5.73 |
|  |  | 63, -33, 42 |  | 5.50 |
|  |  |  |  |  |
| Superior Frontal Cortex | Right | 21, 48, 0 | 55 | 8.27 |
|  |  |  |  |  |
| Inferior Parietal Cortex | Left | -51, -48, 54 | 99 | 7.96 |
|  |  | -42, 63, 54 |  | 6.85 |
|  |  | -54, -39, 54 |  | 6.43 |
|  |  |  |  |  |
| Cuneus/Precuneus/IPC | Right | 21, -84, 45 | 46 | 7.52 |
|  |  | 30, -87, 36 |  | 6.67 |
|  |  | 9, -84, 45 |  | 5.06 |
|  |  |  |  |  |
| Cerebellum/Lingual | Right | 30, -81, -18 | 85 | 6.38 |
|  |  | 45, -69, -24 |  | 6.31 |
|  | Left | -12, -84, -15 | 170 | 6.16 |
|  |  | -6, -81, -9 |  | 4.99 |
|  |  | -33, -81, -21 |  | 4.85 |
| Precentral Gyrus | Left | -30, -12, 60 | 59 | 4.68 |
|  |  | -24, -9, 72 |  | 4.00 |
|  |  | -18, -15, 60 |  | 3.85 |
|  |  |  |  |  |

Note: Parahipp = parahippocampal; Sup Parietal = superior parietal; AI = anterior insula; AMY = amygdala; vmPFC = ventromedial prefrontal cortex; SMA = supplementary motor area; IPC = inferior parietal cortex

Whole-brain t-scores in this table were cluster-thresholded at p < .001, to equate to p < .05, FWE. Italicized regions indicate whole-brain clusters that overlapped with ROI regions.
