# Supplementary material for: Capacity for upregulation of emotional processing in psychopathy: all you have to do is ask
Source: Soc Cogn Affect Neurosci. 2018 Sep 25;13(11):1163–76. doi: 10.1093/scan/nsy088 (PMC6234320; doi:10.1093/scan/nsy088)
Supplement: Supplementary Data [file nsy088_suppl_data.zip › scan-17-477-File017.docx]

Table s10. Regions showing differential activity between Neg_WATCH_ and Neut_WATCH_ trials in High Psychopathy Group.

| **Region** | **L/R** | **Peak coordinate** | **Cluster size** | **t-score** |
| --- | --- | --- | --- | --- |
| *Neg_WATCH_ > Neut_WATCH_* | | | | |
|  |  |  |  |  |
| Inferior Occipital Cortex | Right | 51, -63, -9 | 42 | 4.05 |
|  |  |  |  |  |
|  |  |  |  |  |
| **Amygdala** | **Right** | **24, 0, -24** | **-** | **2.76** |
|  | **Left** | **-21, -9, -12** | **-** | **2.75** |
|  |  |  |  |  |
| *Neut_WATCH_ > Neg_WATCH_* |  |  |  |  |
|  |  |  |  |  |
| Fusiform/Parahippocampal | Right | 30, -33, -18 | 200 | 4.84 |
|  |  | 33, -45, -6 |  | 4.37 |
|  | Left | -27, -42, -12 | 136 | 4.81 |
|  |  |  |  |  |
| Occipital Cortex | Bilateral | -15, -90, -3 | 138 | 4.59 |
|  |  | 6, -90, 0 |  | 3.87 |
|  |  | -6, -75, -6 |  | 3.19 |
|  |  |  |  |  |
| Lingual/Vermis/Calcarine | Right | 12, -54, 12 | 186 | 4.49 |
|  |  | 21, -60, 27 |  | 4.43 |
|  | Left | --9, 54, 9 | 143 | 4.11 |
|  |  | -18, 60, 21 |  | 4.07 |
|  |  |  |  |  |
| Superior/Middle Frontal Cortex | Right | 15, 54, -3 | 62 | 4.42 |
|  |  | 27, 63, 3 |  | 3.32 |
|  |  |  |  |  |
| Superior Temporal Cortex |  | -36, -54, 21 | 40 | 3.80 |
|  |  | -48, -48, 15 |  | 3.23 |
|  |  | 6, -39, 60 |  | 3.69 |
|  |  |  |  |  |

Whole-brain t-scores in this table were cluster-thresholded at p < .001, to equate to p < .05, FWE. Italicized regions indicate whole-brain clusters that overlapped with ROI regions. Where overlap did not occur, small-volume correction was initiated within 10mm ROI spheres, and thresholded at *p* < .05, FWE-svc (bolded).
