# Supplementary material for: Capacity for upregulation of emotional processing in psychopathy: all you have to do is ask
Source: Soc Cogn Affect Neurosci. 2018 Sep 25;13(11):1163–76. doi: 10.1093/scan/nsy088 (PMC6234320; doi:10.1093/scan/nsy088)
Supplement: Supplementary Data [file nsy088_suppl_data.zip › scan-17-477-File008.docx]

Table s1. Regions showing increased activity to negative pictures (ie. all Neg_WATCH_, Neg_INCREASE_, Neg_DECREASE_ trials) compared to neutral pictures (ie. Neut_WATCH_ trials)

| **Region** | **L/R** | **Peak coordinate** | **Cluster size** | **t-score** |
| --- | --- | --- | --- | --- |
| *Negative Pictures > Neutral Pictures* | | | | |
|  |  |  |  |  |
| *Occipital/Cerebellum* | Bilateral | 48, -63, -9 | 3496 | 10.98 |
|  |  | -45, -72, -6 |  | 9.38 |
|  |  | -42, -51, -18 |  | 8.45 |
|  |  |  |  |  |
| *OFC/AI/AMY/ACC/MPFC/vmPFC* | Bilateral | 9, 0 – 3 | 8643 | 7.73 |
|  |  | *-48, 15, 9* |  | *7.60* |
|  |  | 15, 6, -3 |  | 7.53 |
|  |  |  |  |  |
| Inferior Parietal/TPJ | Left | -42, -39, 42 | 1283 | 6.57 |
|  |  | -60, -45, 36 |  | 6.33 |
|  |  | -45, -57, 51 |  | 6.33 |
|  |  |  |  |  |
| Mid/Posterior Cingulate Cortex | Bilateral | 0, -15, 39 | 349 | 5.90 |
|  |  | -6, -51, 30 |  | 3.98 |
|  |  | -3, -39, 24 |  | 3.91 |
|  |  |  |  |  |
| Inferior Parital/TPJ | Right | 42, -39, 45 | 444 | 5.17 |
|  |  | 36, -54, 57 |  | 5.14 |
|  |  |  |  |  |
| Brainstem | Bilateral | -3, -36, -51 | 33 | 3.67 |
|  |  | 6, -36, -51 |  | 3.67 |
|  |  |  |  |  |
|  |  |  |  |  |
| *Neutral Pictures > Negative Pictures* |  |  |  |  |
|  |  |  |  |  |
| Lingual/Vermis/Calcarine | Bilateral | 15, -54, 15 | 2498 | 11.95 |
|  |  | -27, -42, -12 |  | 11.39 |
|  |  | 30, -39, -12 |  | 11.21 |
|  |  |  |  |  |
| Precuneus | Left | -39, -78, 27 | 110 | 5.64 |
|  |  |  |  |  |
| Superior Temporal Cortex | Right | 57, -6, -6 | 572 | 5.22 |
|  |  | 66, -18, 6 |  | 5.18 |
|  |  | 45, -27, 15 |  | 4.82 |
|  | Left | -51, -30, 9 | 285 | 4.41 |
|  |  | -57, -21, 3 |  | 4.21 |
|  |  |  |  |  |
| Postcentral Gyrus | Right | 9, -51, 51 | 313 | 4.99 |
|  |  | 6, -36, 60 |  | 4.53 |
|  |  | 15, -42, 69 |  | 3.78 |
|  |  |  |  |  |
| Postcentral Cortex | Right | 27, -21, 69 | 62 | 3.99 |
|  | Left | -27, -27, 63 | 29 | 3.68 |
|  |  |  |  |  |
|  |  |  |  |  |

Whole-brain t-scores in this table were cluster-thresholded at p < .001, to equate to p < .05, FWE. Italicized regions indicate whole-brain clusters that overlapped with ROI regions.
