# Supplementary material for: Capacity for upregulation of emotional processing in psychopathy: all you have to do is ask
Source: Soc Cogn Affect Neurosci. 2018 Sep 25;13(11):1163–76. doi: 10.1093/scan/nsy088 (PMC6234320; doi:10.1093/scan/nsy088)
Supplement: Supplementary Data [file nsy088_suppl_data.zip › scan-17-477-File018.docx]

Table s11. Regions showing differential activity between Neg_WATCH_ and Neut_WATCH_ trials for High Psychopathy compared to Low/Mid Psychopathy Groups.

| **Region** | **L/R** | **Peak coordinate** | **Cluster size** | **t-score** |  |  |  |  |
| --- | --- | --- | --- | --- | --- | --- | --- | --- |
| *Neg_WATCH_ > Neut_WATCH_ for Low/Mid Psychopathy Groups > High Psychopathy Group* | | | | |  |  |  |  |
|  |  |  |  |  |  |  |  |  |
| Orbital/Superior Frontal Cortex | Left | -39, 57, 0 | 71 | 4.23 |  |  |  |  |
|  |  | -27, 60, 3 |  | 4.11 |  |  |  |  |
|  |  | -48, 45, -6 |  | 4.44 |  |  |  |  |
|  | Right | 21, 57, -6 | 34 | 4.11 |  |  |  |  |
|  |  | 27, 63, 3 |  | 3.24 |  |  |  |  |
|  |  |  |  |  |  |  |  |  |
| Occipital Cortex | Left | -15, -93, -6 | 46 | 4.06 |  |  |  |  |
|  |  |  |  |  |  |  |  |  |
| Angular Cortex | Left | -30, -60, 30 | 67 | 3.90 |  |  |  |  |
|  |  | -36, -54, 21 |  | 3.62 |  |  |  |  |
|  |  |  |  |  |  |  |  |  |
| Inferior Parietal Cortex | Left | -30, -48, 45 | 73 | 3.85 |  |  |  |  |
|  |  | -45, -45, 45 |  | 3.51 |  |  |  |  |
|  |  | -33, -60, 57 |  | 3.18 |  |  |  |  |
|  |  |  |  |  |  |  |  |  |
| *Neg_WATCH_ > Neut_WATCH_ for High Psychopathy Group > Low/Mid Psychopathy Groups* | | | | |  |  |  |  |
|  |  |  |  |  |  |  |  |  |
| *No significant activations.* | | | | |  |  |  |  |
|  | | | | |  |  |  |  |

Whole-brain t-scores in this table were cluster-thresholded at p < .001, to equate to p < .05, FWE.
