# Supplementary figures and images for: Capacity for upregulation of emotional processing in psychopathy: all you have to do is ask
Source: Soc Cogn Affect Neurosci. 2018 Sep 25;13(11):1163–76. doi: 10.1093/scan/nsy088 (PMC6234320; doi:10.1093/scan/nsy088)

Figure s4. NegWATCH > NeutWATCH pooled effects


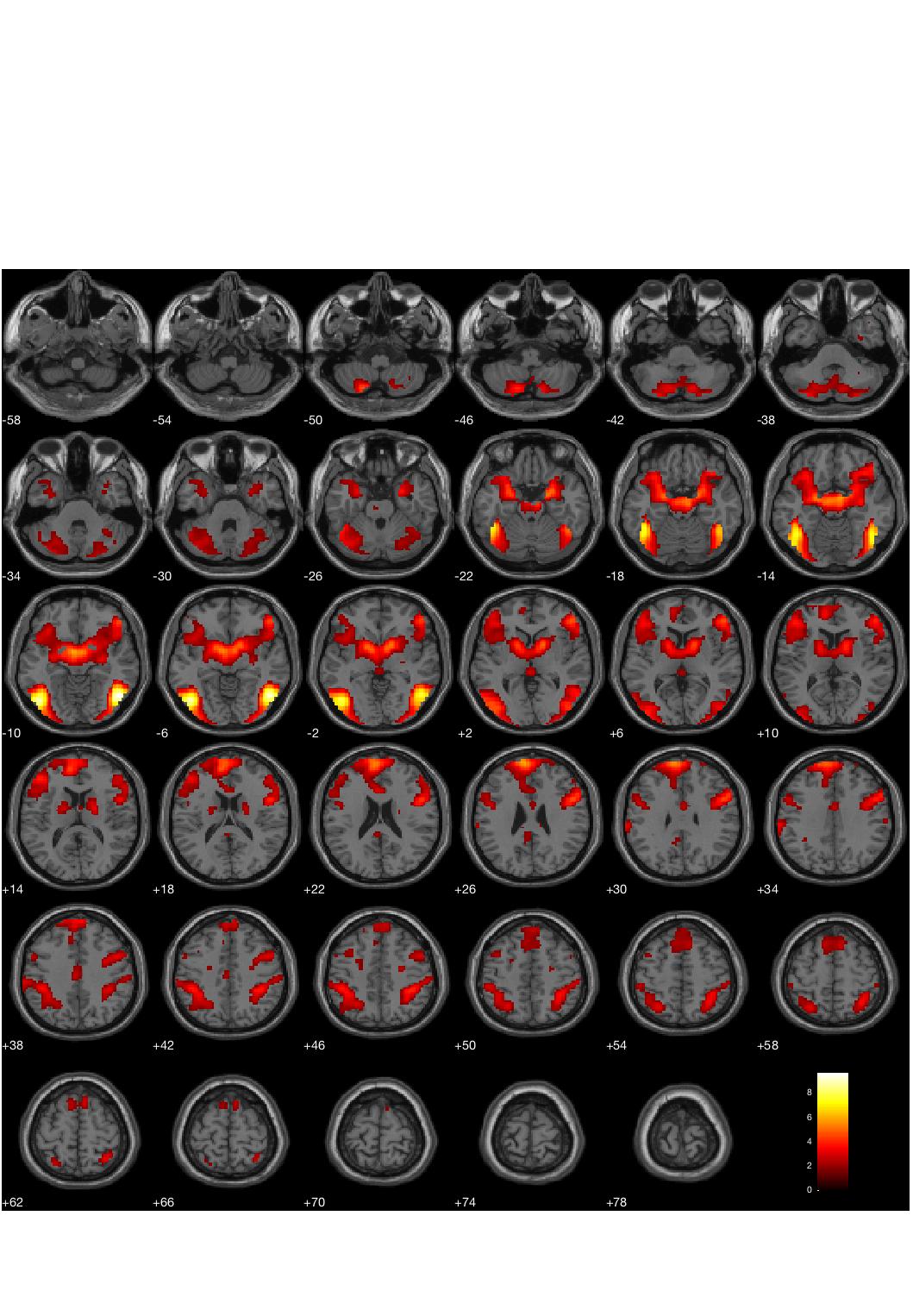


Note: image is shown at the .05 significance level.

Supplement: Supplementary Data [file nsy088_suppl_data.zip › scan-17-477-File031.docx]

Figure s5. NegINCREASE > NegWATCH pooled effects


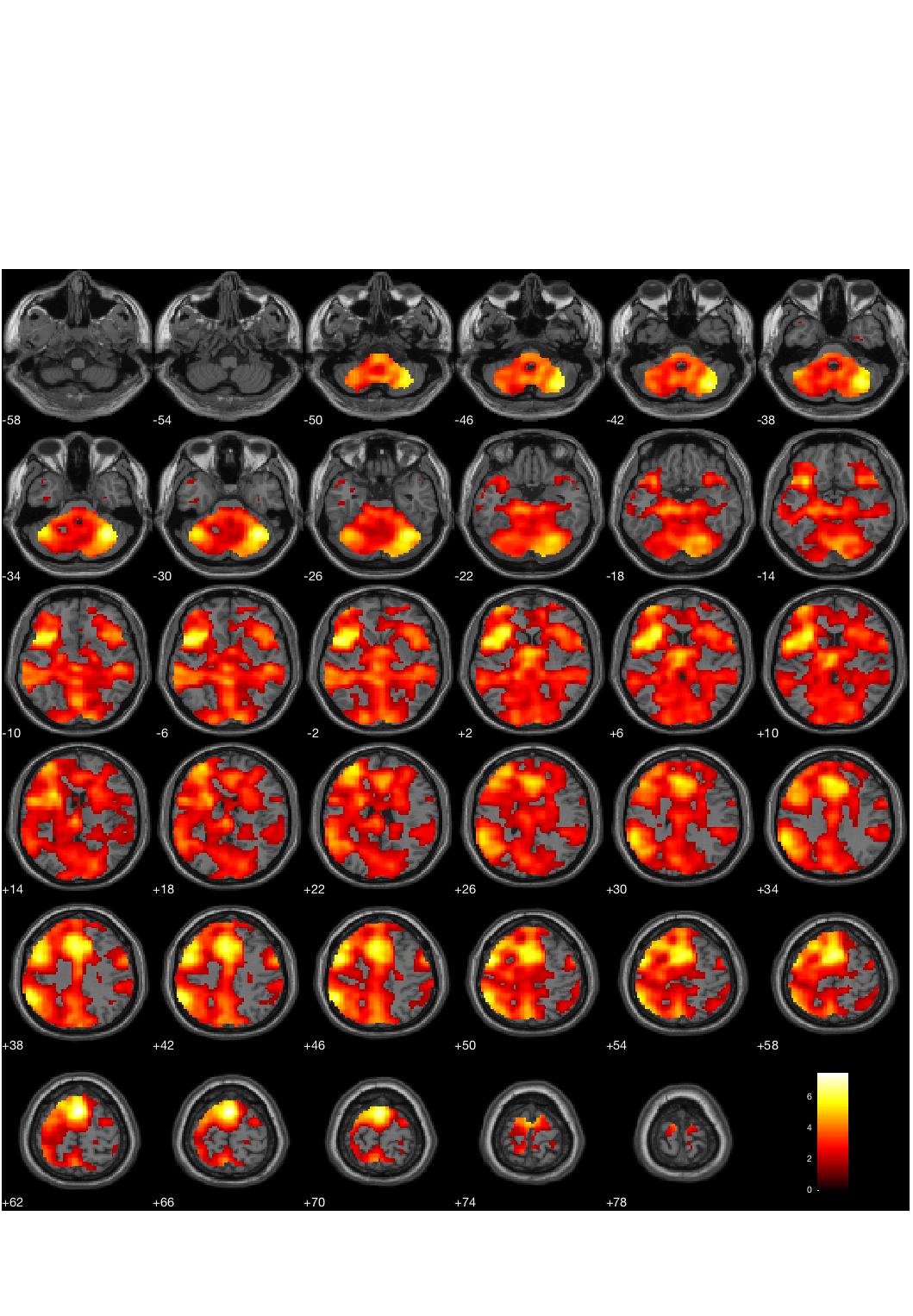


Note: image is shown at the .05 significance level.

Supplement: Supplementary Data [file nsy088_suppl_data.zip › scan-17-477-File032.docx]

Figure s3. Group x TrialType Interaction


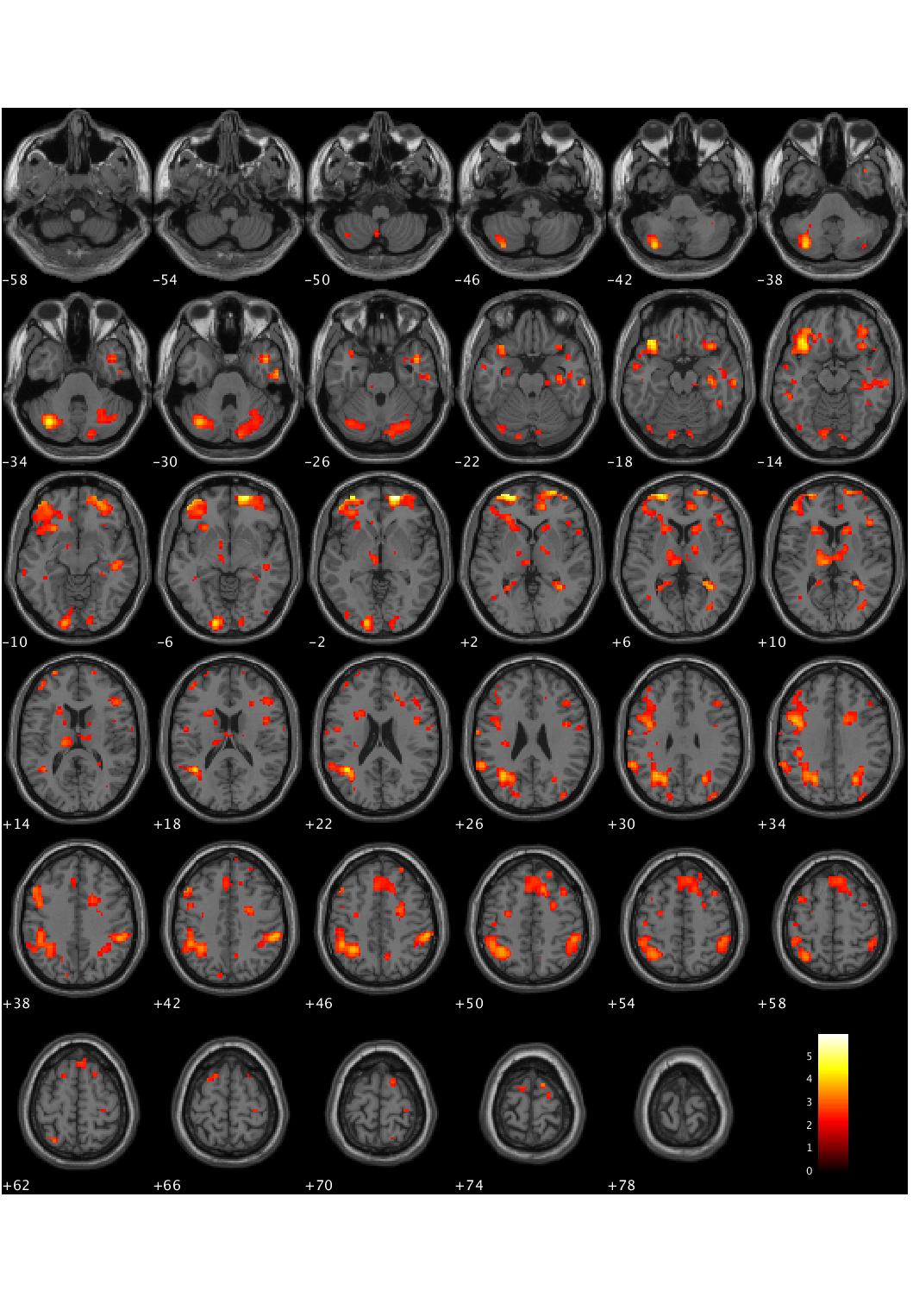


Note: image is shown at the .05 significance level.

Supplement: Supplementary Data [file nsy088_suppl_data.zip › scan-17-477-File030.docx]

Figure s6. NegDECREASE > NegWATCH pooled effects


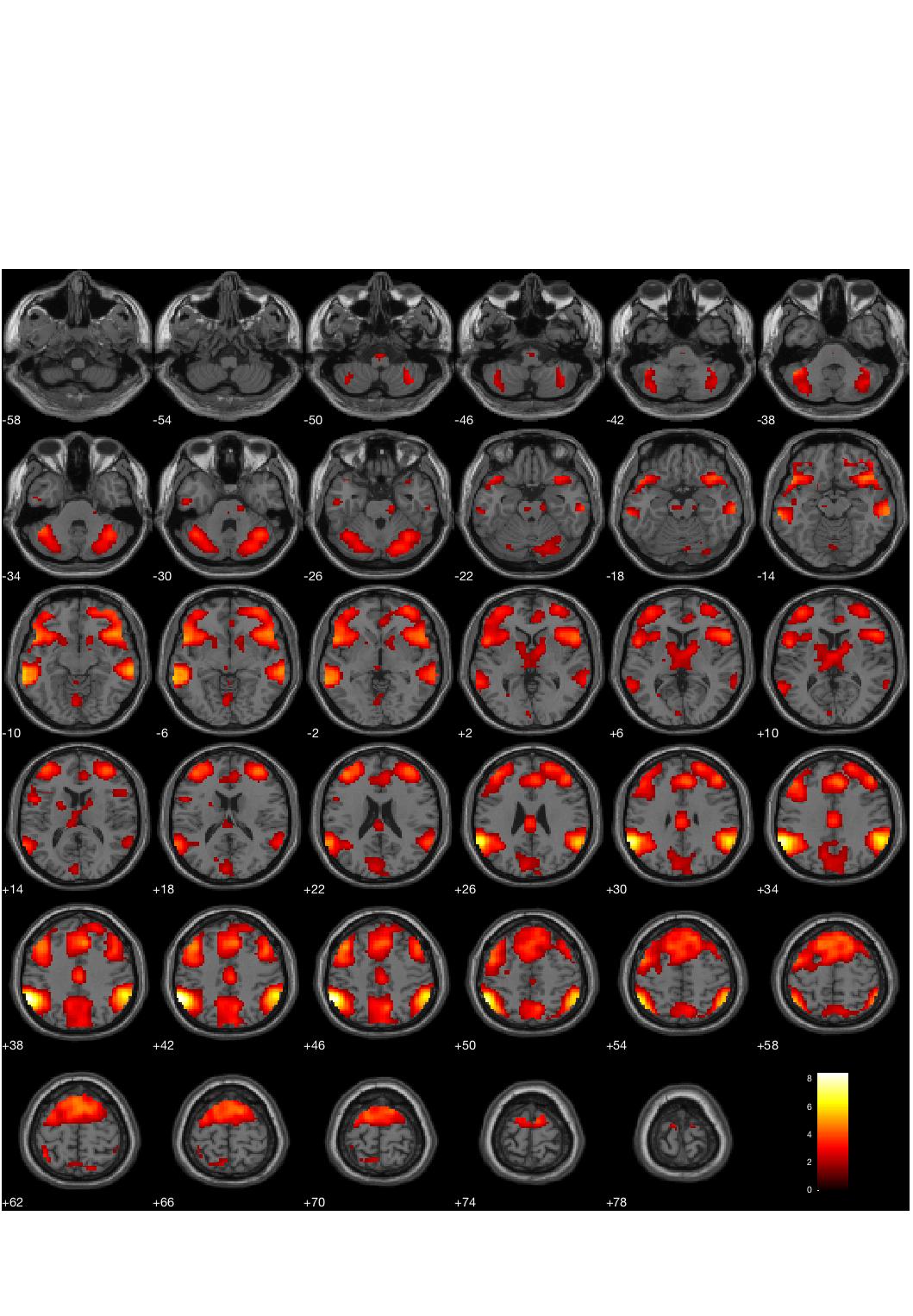


Note: image is shown at the .05 significance level.

Supplement: Supplementary Data [file nsy088_suppl_data.zip › scan-17-477-File033.docx]

Figure s2. Main Effect of Group


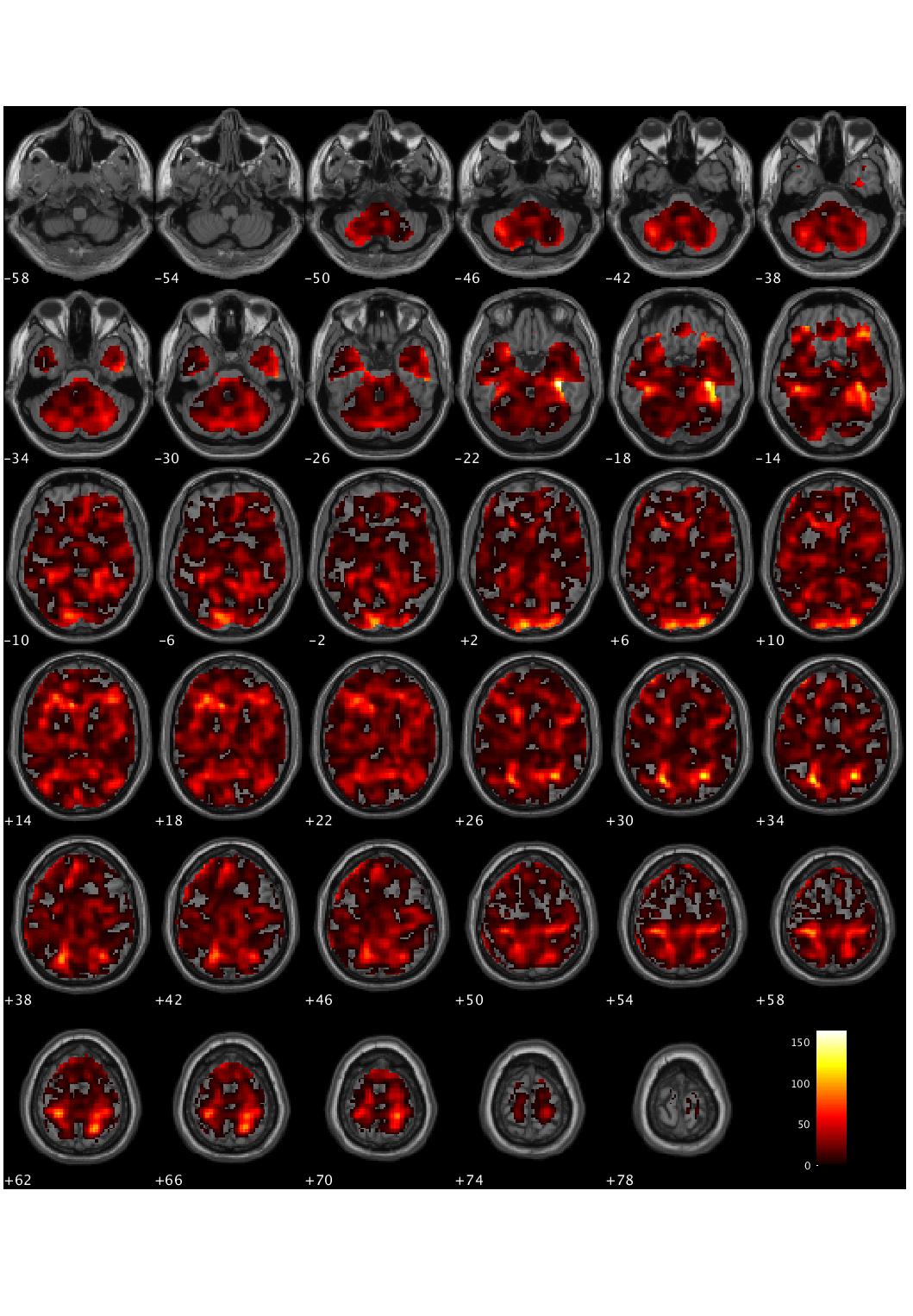


Note: image is shown at the .05 significance level.

Supplement: Supplementary Data [file nsy088_suppl_data.zip › scan-17-477-File029.docx]

Figure s1. Main Effect of Trial Type
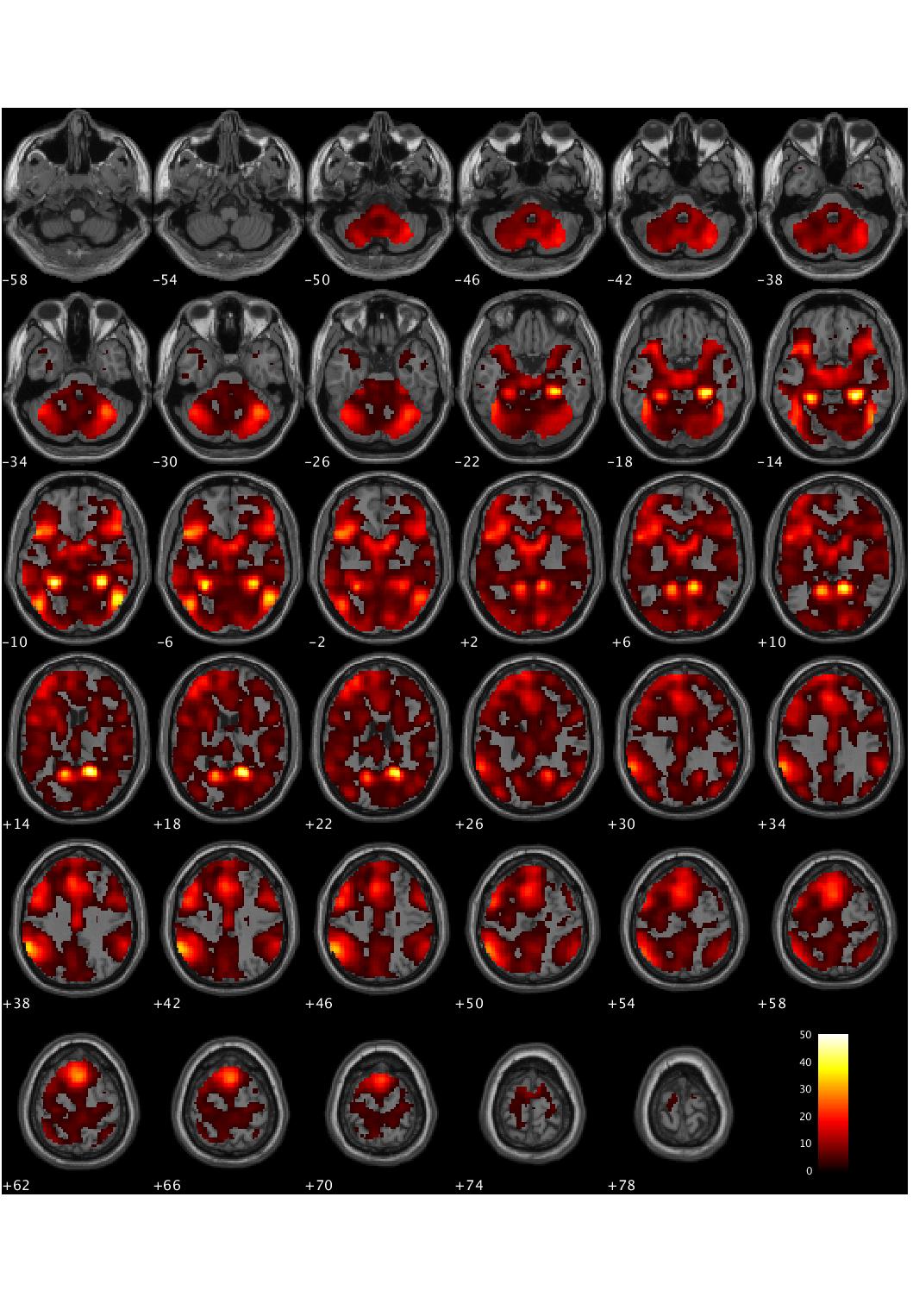


Note: image is shown at the .05 significance level.

Supplement: Supplementary Data [file nsy088_suppl_data.zip › scan-17-477-File028.docx]
